# Multi-Mycotoxin Contamination of Aquaculture Feed: A Global Survey

**DOI:** 10.3390/toxins17030116

**Published:** 2025-03-01

**Authors:** Christiane Gruber-Dorninger, Anneliese Müller, Roy Rosen

**Affiliations:** 1 dsm-firmenich, Animal Nutrition and Health R&D Center Tulln, Technopark 1, 3430 Tulln, Austria; roy.rosen@dsm-firmenich.com; 2 dsm-firmenich, ANH Performance Solutions, Erber Campus 1, 3131 Getzersdorf, Austria

**Keywords:** mycotoxin, deoxynivalenol, fumonisin, zearalenone, aflatoxin, fish, shrimp, aquaculture, feed

## Abstract

Plant-based materials are increasingly being used as ingredients of aquaculture feed. These materials are prone to mycotoxin contamination, as mycotoxigenic fungi infest crop plants in the field and agricultural products during storage. As mycotoxins can cause toxic effects in aquatic animals, their occurrence in feedstuffs should be monitored. To this end, we performed an extensive global survey of mycotoxin contamination in aquaculture feed and plant-based feed raw materials. We collected samples of compound feed for fish (n = 226) and shrimps (n = 61), maize (n = 3448), maize DDGS (n = 149), wheat (n = 1578), soybean (n = 428), and rice (n = 65). We analyzed concentrations of 51 mycotoxins, emerging mycotoxins, masked mycotoxins, and mycotoxin metabolites. Mycotoxins were almost ubiquitously present in compound feed, as >90% of samples were contaminated with at least one mycotoxin. Feed raw materials exhibited distinct mycotoxin occurrence patterns consistent with known susceptibility to fungal pathogens and with their production process. Unsafe concentrations of aflatoxin B_1_ exceeding the EU maximum level were detected in 7.2% of fish feed samples. While most feedstuffs complied with EU guidance values for deoxynivalenol, zearalenone, and fumonisins, a comparison of detected concentrations with dietary concentrations reported to cause adverse effects in fish and shrimps in published studies indicated that significant fractions of samples contained potentially harmful levels of these mycotoxins. In addition to regulated mycotoxins, several emerging mycotoxins (e.g., enniatins, beauvericin, alternariol, moniliformin) were prevalent. Feed was frequently co-contaminated with multiple mycotoxins indicating a risk of combined effects. In conclusion, mycotoxin contamination was common in aquaculture feed and fractions of samples were contaminated with mycotoxin levels known to exert adverse effects in aquaculture species. Results of this survey highlight the necessity for targeted studies on the effects of frequently detected mycotoxin mixtures and emerging mycotoxins in fish and shrimp.

## 1. Introduction

The world’s population is growing rapidly and is predicted to be more than 10 billion people in the coming century [1]. To ensure this growing population has adequate food supply and protein in particular, major changes are taking place in the way human food and farm animal feed are produced. In the case of aquatic foods, global aquaculture investments and production have seen rapid growth. Aquatic food consumption has increased significantly and will continue to rise [2] and aquaculture production must continue to increase as well, in order to supply the amounts required for the growing global population [3].

Like in the farming of terrestrial animals, the feed used in intensive and semi-intensive aquaculture practices has great bio-economic importance. Therefore, aquaculture feeds and the raw ingredients used for their production are constantly evolving. In recent years, this evolution has been influenced by socioeconomic and environmental factors. Perhaps the most notable development is the reduction in the long-standing reliance on raw materials of marine origin, i.e., fish meals and fish oils. This conceptual change has led to an increased use and development of plant-sourced and emerging alternative protein and oil products. Even though the dietary needs vary between aquatic species, increased inclusion rates, and in some cases, the complete substitution of marine ingredients by plant-based and alternative raw ingredients has been successfully demonstrated [4,5,6,7]. Moreover, these formulation adaptations have been commercially implemented to various extents for most major farmed species. While this shift to plant-based diets is a positive development from an environmental and economic point of view, it has also introduced new challenges due to the presence of mycotoxins and other anti-nutritional factors in plant-based feed. Mycotoxins are chemically and thermally stable molecules and consequently show a high degree of stability during food and feed processing. In general, processes that use the highest temperatures have the greatest effect [8,9]. However, even the process of extrusion, which involves the application of high temperatures and pressure and is commonly used, among other reasons, to reduce microbial and parasite contamination in aquaculture feed, reduces mycotoxin contamination only to a certain extent [8,9,10,11].

Mycotoxins are “natural products produced by fungi that evoke a toxic response when introduced in low concentration to higher vertebrates and other animals by a natural route” as defined by Bennett [12]. Fungal infestation and mycotoxin production can occur during the growth of crop plants in the field or during storage of harvested crops [13,14]. A group of mycotoxins are considered “major mycotoxins” due to frequent occurrence and severity of toxic effects [15]. This group includes aflatoxins, deoxynivalenol (DON), fumonisins, zearalenone (ZEN), T-2 toxin, ochratoxin A (OTA), and certain ergot alkaloids. These mycotoxins differ in their molecular structures, biological modes of action, and the toxicological effects they elicit. For example, aflatoxins are known for their hepatotoxic effect and carcinogenicity [16], DON inhibits protein biosynthesis and causes feed refusal [17], fumonisins inhibit the sphingolipid metabolism [18,19], and ZEN is an estrogenic mycotoxin that interferes with reproductive health [20,21]. The effects of these mycotoxins are increasingly studied and described in terrestrial farmed animals and to a smaller extent also in aquaculture species. Many countries have stipulated regulatory limits or guidance values for mycotoxins in animal feed. For example, in the European Union (EU), maximum levels are enforced for aflatoxin B_1_ (AFB_1_) in feed [22], and guidance values have been stipulated for DON, ZEN, the sum of fumonisins B_1_ and B_2_ (FB_1_ + FB_2_), and OTA [23,24].

In addition to the major mycotoxins, plant pathogenic fungi produce a plethora of other secondary metabolites that are less well investigated. Compounds such as beauvericin, enniatins, moniliformin, alternariol, tenuazonic acid, and sterigmatocystin are often referred to as “emerging mycotoxins”, i.e., these mycotoxins are not regulated or routinely monitored, yet evidence of their widespread occurrence has been emerging recently [25,26]. Besides emerging mycotoxins, animal feed may also be contaminated with mycotoxin derivatives formed by fungi (e.g., 3-acetyl-DON), or “masked mycotoxins”, i.e., mycotoxin conjugates formed by the natural defense system of the host plant (e.g., DON-3-glucoside) [27,28]. It should be noted that most fungi produce multiple mycotoxins, and agricultural commodities are often infested with multiple fungal strains. Therefore, the effects of mycotoxin mixtures in animals deserve scientific attention, as the effects of mixtures are often greater than the effects of the individual mycotoxins [29,30]. There are currently significant knowledge gaps on the occurrence of emerging mycotoxins, masked mycotoxins, and mycotoxin mixtures in animal feed as well as on their effects in animals and particularly aquatic animal species.

According to a recent survey, the global prevalence of major mycotoxins in food and feed crops was 60–80%, and 20–25% of crops were contaminated above EU and Codex Alimentarius limits for major mycotoxins in food [31]. Furthermore, in a large-scale global survey of mycotoxin contamination in animal feed performed from 2008 to 2017, 88% of samples were contaminated with at least one major mycotoxin [32]. These data indicate a high global prevalence of major mycotoxins. However, concerning aquaculture feeds and the main raw ingredients used by the aquaculture industry, limited data are available on both the metrics of mycotoxin contamination and the risk it brings to the sector. A survey of major mycotoxin occurrence in compound aquaculture feed from Europe and Asia performed in 2014 found high occurrence and co-occurrence levels, as well as high average mycotoxin concentrations [33]. Similar observations were made in a survey of fish feeds and raw ingredients from European countries performed from 2012 to 2019 [34].

In this study, we performed a global survey of mycotoxin contamination in fish and shrimp feed, as well as plant-based feed raw materials. We analyzed the concentrations of 51 mycotoxins, emerging mycotoxins, and mycotoxin metabolites using liquid chromatography tandem mass spectrometry (LC-MS/MS) analysis. Furthermore, we analyzed mycotoxin co-occurrence and determined the most prevalent mycotoxin mixtures. To characterize the risk of mycotoxin contamination in feed to fish and shrimps, we compared the detected concentrations to maximum levels and guidance values that are in effect in the EU and to dietary concentrations shown to exert adverse effects in fish and shrimps in published studies. This comprehensive dataset on global multi-mycotoxin occurrence in aquaculture feed provides a basis for risk assessment and for the prioritization of research efforts on toxicological effects of mycotoxins and mycotoxin mixtures in fish and shrimps.

## 2. Results

### 2.1. Mycotoxin Occurrence in Compound Feed Destined for Fish and Shrimps and Comparison to EU Limits

**Figure 1:**
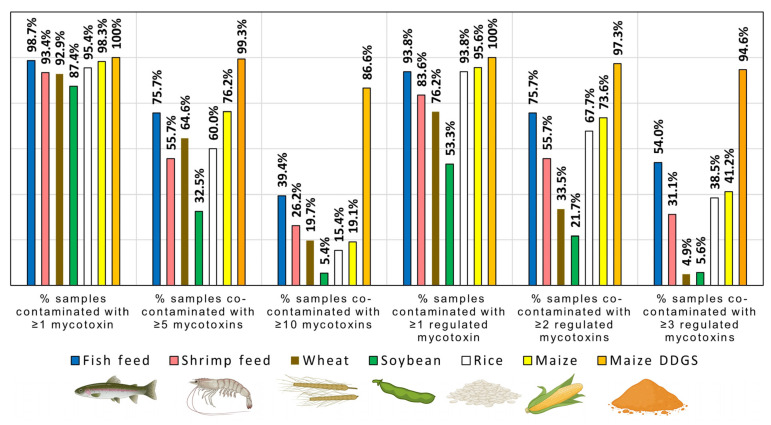
Overview of mycotoxin occurrence and co-occurrence in compound feed and feed raw materials. The figure shows percentages of samples contaminated with ≥1, ≥5, or ≥10 of the 51 mycotoxins and fungal metabolites analyzed in this study, as well as percentages of samples contaminated with ≥1, ≥2, or ≥3 mycotoxins that are subjected to an EU maximum level (i.e., aflatoxin B_1_) or guidance value (i.e., deoxynivalenol, zearalenone, ochratoxin A, and the sum of fumonisins B_1_ and B_2_). Illustrations of animals and feed raw materials were created in Biorender.

Mycotoxins were almost ubiquitously present in compound feed destined for fish and shrimps. In the case of fish feed and shrimp feed, 98.7% and 93.4% of the samples, respectively, were contaminated with at least one of the 51 mycotoxins and fungal metabolites analyzed (Figure 1) and in total 46 and 33 mycotoxins and fungal metabolites were detected. Furthermore, 93.8% of fish feed samples and 83.6% of shrimp feed samples (Figure 1) were contaminated with at least one mycotoxin regulated in feed in the EU with either a maximum level (i.e., AFB_1_) or a guidance value (i.e., DON, ZEN, OTA, and FB_1_ + FB_2_).

In fish feed, several of the regulated mycotoxins were prevalent (Table 1). In total, 41.6% of the samples were contaminated with AFB_1_, 68.6% of the samples were contaminated with FB_1_ + FB_2_, 61.1% of the samples were contaminated with ZEN, and 58.0% of the samples were contaminated with DON. A subset of the samples was contaminated with potentially unsafe levels of AFB_1_. In total, 7.1% of the samples did not comply with the EU maximum level for AFB_1_ (i.e., 10 µg/kg; Table 2) and a high 90th percentile value of 93.8 µg/kg was detected (Table 1). In addition to AFB_1_, aflatoxins B_2_, G_1_, and G_2_ were detected in fish feed (Table 1). These aflatoxins were detected in lower fractions of samples than AFB_1_ (i.e., 3.5–12.4%) yet reached relatively high median levels of 3.8–10.5 µg/kg. All of the samples complied with EU guidance values for DON, ZEN, and OTA (Table 2). Only one sample (0.4%) exceeded the guidance value for FB_1_ + FB_2_ (i.e., 10 mg/kg; Table 2).

Shrimp feed samples showed overall lower levels of contamination with regulated mycotoxins compared to fish feed samples (Table 1). In total, 16.4% of the samples were contaminated with AFB_1_, 60.7% of the samples were contaminated with DON, 41.0% of the samples were contaminated with FB_1_ + FB_2_, and 37.7% of the samples were contaminated with ZEN. One of the samples (1.6%) exceeded the EU maximum level for AFB_1_ in shrimp feed (i.e., 10 µg/kg), and all of the samples complied with the EU guidance values for DON, ZEN, FB_1_ + FB_2_, and OTA (Table 2).

In addition to regulated mycotoxins, a variety of unregulated mycotoxins, less investigated fungal metabolites (“emerging mycotoxins”), and mycotoxin metabolites were detected in both fish and shrimp feed (Table 1). The most prevalent emerging mycotoxins were enniatins B and B_1_, as well as beauvericin, alternariol, and moniliformin (detected in 20.8–54.0% of fish feed samples and 8.2–50.8% of shrimp feed samples (Table 1). Furthermore, sterigmatocystin and mycophenolic acid were detected in 16.8% and 11.9% of fish feed samples, as well as 8.2% and 6.6% of shrimp feed samples, respectively. In addition, a variety of ergot alkaloids were detected in both fish and shrimp feed. They were less prevalent and mostly detected in lower concentrations compared to the regulated mycotoxins (Table 1). The DON metabolite DON-3-glucoside was detected in 23.9% and 24.6% of fish and shrimp feed samples, respectively, at lower concentrations than its parent compound (Table 1).

### 2.2. Comparison of Mycotoxin Concentrations in Compound Feed to Harmful Effects Reported in the Literature

To characterize the risk of mycotoxin occurrence in compound feed below EU guidance values, we conducted a literature search to identify published studies that found adverse effects of sub-regulatory dietary DON, ZEN, or fumonisin concentrations in fish and shrimps. In the case of fish, the literature search resulted in 99 hits in Pubmed and 143 hits in Scopus. In total, 160 publications remained after the removal of duplicates, publications written in languages other than English, conference papers, and book chapters. Screening of these 160 publications identified 41 publications that reported adverse effects of sub-regulatory dietary DON, ZEN, or fumonisin concentrations in fish (Table 3, Table 4 and Table 5). In the case of shrimps, the literature search resulted in 24 hits in Pubmed and 39 hits in Scopus. In total, 39 publications remained after the removal of duplicates, publications written in languages other than English, conference papers, and book chapters. Of these publications, five were found to report adverse effects of sub-regulatory dietary DON or fumonisin concentrations in shrimps (Table 3 and Table 5). No publication reported adverse effects of sub-regulatory dietary ZEN in shrimps.

**Figure 2:**
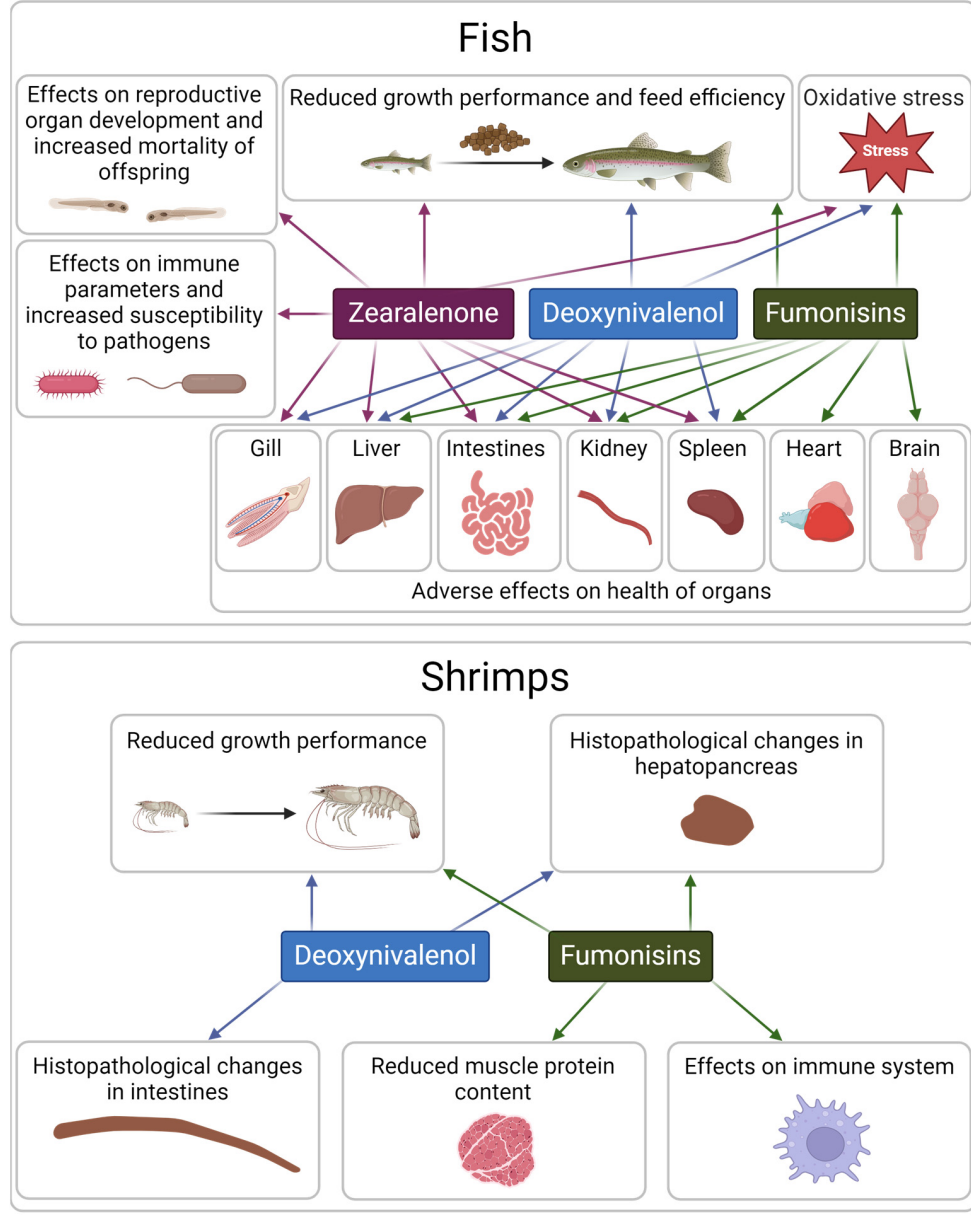
Adverse effects of dietary deoxynivalenol, zearalenone, and fumonisin concentrations at or below EU guidance values in fish and shrimps. For more details on the depicted effects please refer to Table 3, Table 4 and Table 5. This figure was created in BioRender (https://BioRender.com/i77b545; accessed on 10 December 2024).

According to published studies, dietary DON concentrations below the EU guidance value (5 mg/kg) negatively affected growth performance and feed efficiency and impaired liver, gut, kidney, gill, and spleen health in salmonids and cyprinids (Table 3; Figure 2). Furthermore, sub-regulatory concentrations of DON compromised the growth performance of shrimps and caused histopathological changes in intestines and hepatopancreas. The lowest observed adverse effect level for DON in fish in these studies was 0.318 mg/kg feed shown to decrease feed intake and cause oxidative stress in grass carp (*Ctenopharyngodon idella*) [35,36,37]. In the case of shrimps, a lowest observed adverse effect level of 0.2 mg/kg DON in feed was detected for decreased growth rate in Pacific white shrimp (*Litopenaeus vannamei*) [38]. In our survey, 9.3% of fish feed samples exceeded 0.318 mg/kg DON and 8.2% of shrimp feed samples exceeded 0.2 mg/kg DON (Table 6) and could, therefore, compromise the health and growth of fish and shrimps.

Dietary ZEN concentrations below the EU guidance value (2 mg/kg) differentially affected growth performance of rainbow trout (*Oncorhynchus mykiss*) in published studies (Table 4). While a dietary concentration of 0.3 mg/kg ZEN administered for 60 days decreased final body weight, weight gain, and specific growth rate and increased the feed conversion ratio [39], a concentration of 1.81 mg/kg ZEN administered for 71 days did not affect growth performance [40] and a concentration of 2 mg/kg administered for 96 weeks increased growth and decreased the feed conversion ratio [41]. A decrease in body weight gain and feed efficiency has also been observed in grass carp upon administration of 1.041–2.507 mg/kg ZEN for 70 days [42]. Furthermore, a decrease in body weight gain was observed in European sea bass upon administration of 0.725 mg/kg ZEN for 28 days [43]. Other effects of sub-regulatory ZEN concentrations in fish included morphological anomalies in gonads, increased mortality of offspring, increased susceptibility to pathogens, oxidative stress, impaired gut health, trunk kidney inflammation, and impaired gill and liver health (Table 4; Figure 2). The lowest observed adverse effect level for ZEN in fish was 0.3 mg/kg feed, which impaired growth performance and gut health in rainbow trout [39]. In our survey, 2.7% of fish feed samples exceeded this concentration (Table 6) and could, therefore, cause negative effects in fish.

Dietary fumonisin concentrations at or below the EU guidance value (10 mg/kg) impaired growth performance and feed conversion in rainbow trout, common carp (*Cyprinus carpio*), and silver catfish (*Rhamdia quelen*) (Table 5). In addition, fumonisins caused alterations in different organs including brain, liver, intestines, pancreas, kidney, heart, and spleen (Table 5; Figure 2). The lowest observed adverse effect level in these studies was 6.2 mg FB_1_/kg feed. This concentration was reported to affect growth performance, as well as spleen and brain health in silver catfish [44,45]. In our survey, 0.4% of fish feed samples (1 sample) exceeded this concentration (Table 6), the same as for the guidance value of 10 mg/kg (Table 2). In Pacific white shrimp, sub-regulatory dietary FB_1_ concentrations negatively affected growth performance, muscle protein content and properties, hepatopancreas health, and components of the immune system (Table 5; Figure 2). A low dietary FB_1_ level of ≥0.2 mg/kg decreased muscle protein content, and ≥ 0.6 mg/kg decreased weight gain [46]. In our survey, 4.9% of shrimp feed samples exceeded 0.2 mg/kg FB_1_ (Table 6) and are, therefore, potentially harmful for shrimps.

**Table 3:** Adverse effects of dietary deoxynivalenol concentrations at or below the EU guidance value (5 mg/kg) in fish and shrimps.

Publication	Species	Trial Duration	Effects of Concentrations at or Below Guidance Values ^a^	Dosages Tested (in mg DON/kg Feed) ^a,b^
Koletsi, et al., 2022 [47]	*Oncorhynchus mykiss*	56 days (42 days restrictive feeding + 14 days ad libitum feeding)	Restrictive feeding: 1.192–1.566 mg/kg DON reduced body weight and protein retention efficiency and induced histopathological changes in the liver. Ad libitum feeding: 1.192–1.566 mg/kg DON reduced growth and specific growth rate and induced histopathological changes in the liver; 1.192 mg/kg DON reduced final body weight; 1.566 mg/kg DON increased feed conversion ratio.	(1) 0.07 mg/kg (control) (2) 0.679 mg/kg (3) 1.192 mg/kg (4) 0.781 mg/kg (5) 1.566 mg/kg (concentrations in dry matter)
Koletsi, et al., 2023 [48]	*Oncorhynchus mykiss*	56 days (42 days restrictive feeding + 14 days ad libitum feeding)	Restrictive feeding: DON (2.809 mg/kg) inhibited growth, reduced final body weight and feed conversion, and decreased protein and energy retention. Ad libitum feeding: DON (2.8 mg/kg) reduced feed intake, growth, and final body weight and increased FCR. DON induced histopathological changes in liver and intestines.	(1) Uncontaminated control (DON not detected) (2) 2.809 mg/kg in dry matter
Koletsi, et al., 2023 [49]	*Oncorhynchus mykiss*	56 days (42 days restrictive feeding + 14 days ad libitum feeding)	Restrictive feeding: 1.209 mg/kg DON in fishmeal-based feed and 1.329 mg/kg DON in soybean meal-based feed increased FCR and decreased final body weight, growth (g/day), specific growth rate, retained protein, protein retention efficiency and retained energy. A total of 1.209 mg/kg DON in fishmeal-based feed decreased energy retention efficiency. Ad libitum feeding: 1.209 mg/kg DON in fishmeal-based feed and 1.329 mg/kg DON in soybean meal-based feed increased FCR and decreased final body weight, growth (g/day) and hepatosomatic index. At different timepoints during the trial, 1.329 mg/kg DON in soybean meal-based feed decreased mucosal fold width and enterocyte width in the midgut.	Fishmeal-based feed: (1) Uncontaminated control (DON not detected) (2) 1.209 mg/kg (concentrations in dry matter) Soybean meal-based feed: (1) 0.046 mg/kg (control) (2) 1.329 mg/kg (concentrations in dry matter)
Hooft, et al., 2011 [50]	*Oncorhynchus mykiss*	56 days	Dose-dependent effect of DON (0.3 mg/kg, 0.8 mg/kg, 1.4 mg/kg, 2.0 mg/kg, 2.6 mg/kg): decrease in weight gain, thermal-unit growth coefficient, feed intake, feed efficiency, retained nitrogen, recovered energy, nitrogen retention efficiency, and energy retention. 1.4–2.6 mg/kg DON caused histopathological changes in the liver.	(1) 0.3 mg/kg (control) (2) 0.8 mg/kg (3) 1.4 mg/kg (4) 2.0 mg/kg (5) 2.6 mg/kg
Hooft and Bureau, 2017 [51]	*Oncorhynchus mykiss*	84 days	Dose-dependent effect of DON (0.3 mg/kg, 1 mg/kg, 1.5 mg/kg, 2.0 mg/kg): decrease in weight gain, feed intake, thermal-unit growth coefficient, whole body crude protein, lipid, ash, gross energy content, retained nitrogen, recovered energy, nitrogen retention efficiency, and energy retention efficiency. Dose-dependent increase in whole body water content.	(1) 0.3 mg/kg (control) (2) 1 mg/kg (3) 1.5 mg/kg (4) 2.0 mg/kg
Hooft et al., 2019 [52]	*Oncorhynchus mykiss*	56 days	Dose-dependent effect of (i) purified DON (0 mg/kg, 0.7 mg/kg, 1.4 mg/kg, and 2.1 mg/kg) and (ii) DON from natural contamination (0 mg/kg, 2.1 mg/kg, 4.1 mg/kg, and 5.9 mg/kg): decrease in weight gain, thermal-unit growth coefficient, feed intake, feed efficiency, retained nitrogen, recovered energy, and nitrogen retention efficiency. In addition, naturally contaminated feed decreased energy retention efficiency and affected chemical body composition.	First set of diets (contamination achieved by addition of purified DON): (1) Uncontaminated control (DON not detected); (2) 0.7 mg/kg; (3) 1.4 mg/kg; (4) 2.1 mg/kg Second set of diets (contamination achieved by addition of naturally contaminated corn): (1) Uncontaminated control (DON not detected); (2) 2.1 mg/kg; (3) 4.1 mg/kg; (4) 5.9 mg/kg
Hooft et al., 2019 [53]	*Oncorhynchus mykiss*	70 days	Dose-dependent effect of DON (~0.1 mg/kg, ~0.7 mg/kg, and ~1.3 mg/kg) regardless of the dietary digestible starch content: decrease in weight gain, thermal-unit growth coefficient, feed efficiency, whole body crude protein content, retained nitrogen, and nitrogen retention efficiency.	First set of diets with 12% digestible starch: (1) 0.148 mg/kg (control); (2) 0.738 mg/kg; (3) 1.246 mg/kg Second set of diets with 24% digestible starch: (1) 0.127 mg/kg (control); (2) 0.750 mg/kg; (3) 1.359 mg/kg
Matejova et al., 2014 [54]	*Oncorhynchus mykiss*	23 days	1.964 mg/kg DON decreased mean cell hemoglobin, glucose, cholesterol, and ammonia levels in the blood and caused histopathological changes in the caudal kidney.	(1) 0.225 mg/kg feed (control) (2) 1.964 mg/kg
Liu et al., 2022 [55]	*Oncorhynchus mykiss*	24 days	5 mg/kg DON reduced mortality following a *Flavobacterium psychrophilum* challenge, increased LC3II protein expression in the liver and modulated the mRNA expression of autophagy-related genes in liver and muscle.	(1) Uncontaminated control (DON concentration not reported) (2) 5 mg/kg
Gonçalves et al., 2018 [56]	*Oncorhynchus mykiss*	50 days	1.166–2.745 mg/kg DON caused histopathological changes in the liver. 2.745 mg/kg DON decreased final weight, specific growth rate, feed intake, and thermal-unit growth coefficient, increased liver enzyme activities in serum, and increased survival rate after *Yersinia ruckeri* challenge.	(1) Uncontaminated control (DON not detected) (2) 1.166 mg/kg (3) 2.745 mg/kg
Gonçalves et al., 2018 [56]	*Oncorhynchus mykiss*	168 days	0.367 mg/kg DON decreased protein efficiency rate and increased feed intake measured on day 62 of the experiment. (A decrease in final body weight was observed with a *p*-value of 0.053.)	(1) Uncontaminated control (DON not detected) (2) 0.367 mg/kg
Gonçalves et al., 2018 [57]	*Oncorhynchus mykiss*	60 days	4.714 mg/kg DON decreased final body weight, specific growth rate, feed intake, hepatosomatic index, and protein efficiency ratio and altered whole body composition and whole-body nutrient retention.	(1) 0.037 mg/kg (control) (2) 4.714 mg/kg
Ryerse et al., 2015 [58]	*Oncorhynchus mykiss*	28 days	3.1 mg/kg DON (purified DON; experiment 1), 3.8 mg/kg DON (purified DON; experiment 2), as well as 3.3 mg/kg DON + 0.5 mg/kg ZEN (natural contamination; experiment 2) significantly reduced feed intake and weight gain.	Experiment 1: (1) <0.1 mg/kg (control) (2) 3.1 mg/kg (purified DON) Experiment 2: (1) <0.1 mg/kg (control) (2) 3.3 mg/kg + 0.5 mg/kg ZEN (natural contamination) (3) 3.8 mg/kg DON (purified DON)
Ryerse et al., 2016 [59]	*Oncorhynchus mykiss*	49 days	Dose-dependent effect of DON + ZEN (4.1 mg/kg DON + 0.5 mg/kg ZEN; 5.9 mg/kg DON + 0.6 mg/kg ZEN): reduction in feed intake and reduction in mortality following a *Flavobacterium psychrophilum* challenge.	(1) <0.5 mg/kg (control) (2) 4.1 mg/kg DON + 0.5 mg/kg ZEN (3) 5.9 mg/kg DON + 0.6 mg/kg ZEN
Šišperová et al., 2015 [60]	*Oncorhynchus mykiss*	32 days	1.964 mg/kg DON affected biomarkers of oxidative stress in liver, kidneys, and gill.	(1) 0.225 mg/kg (control) (2) 1.964 mg/kg
Bernhoft et al., 2018 [61]	*Salmo salar*	56 days	Dose-dependent effect of DON (0.5 mg/kg, 1 mg/kg, 2 mg/kg, 4 mg/kg, and 6 mg/kg): decrease in feed efficiency, weight gain, length, condition factor, and antibody response to *Aeromonas salmonicidae* vaccination. Increase in relative liver weight. Various effects on clinical biochemical parameters.	(1) Uncontaminated control (DON not detected) (2) 0.5 mg/kg (3) 1.0 mg/kg (4) 2.0 mg/kg (5) 4.0 mg/kg (6) 6.0 mg/kg
Pietsch et al., 2014 [62]	*Cyprinus carpio* L.	42 days	0.352–0.953 mg/kg DON: leucocytes obtained from head kidneys showed reduced viability and function when cultured in vitro.	(1) Uncontaminated control (DON not detected) (2) 0.352 mg/kg (3) 0.619 mg/kg (4) 0.953 mg/kg
Pietsch et al., 2014 [63]	*Cyprinus carpio* L.	42 days	0.352–0.953 mg/kg DON caused histopathological changes in the liver and increased lactate dehydrogenase activity in kidneys. 0.619–0.953 mg/kg DON increased lipid content and energy content in whole body homogenate. 0.953 mg/kg DON increased lipid peroxidation in liver, head kidney, and spleen.	(1) Uncontaminated control (DON not detected) (2) 0.352 mg/kg (3) 0.619 mg/kg (4) 0.953 mg/kg
Pietsch and Burkhardt-Holm, 2015 [64]	*Cyprinus carpio* L.	56 days	0.953 mg/kg DON increased alanine aminotransferase activity in liver on days 26 and 56, increased glutathione S-transferase activity on day 56, decreased glutathione peroxidases activity on days 26 and 56, decreased superoxide dismutase activity on day 56, and induced histopathological changes on days 14 and 26.	(1) Uncontaminated control (DON not detected) (2) 0.953 mg/kg
Pietsch et al., 2015 [65]	*Cyprinus carpio* L.	56 days	0.953 mg/kg DON fed to, sampling on days 7, 14, 26, 56 of the trial. DON affected differential blood cell counts on days 7 and 14 (but on days 26, 56). mRNA expression of immunity-related genes increased in intestines and spleen on day 14, and in trunk kidney on day 26, but not on other sampling days.	(1) Uncontaminated control (DON not detected) (2) 0.953 mg/kg
Huang et al., 2018 [37]	*Ctenopharyngodon idella*	60 days	0.318–1.515 mg/kg DON decreased feed intake and intestine weight and affected biomarkers of oxidative stress and antioxidant status in the intestines. The effects on mRNA and protein expression of gut proteins suggest tissue damage in intestines. 0.636–1.515 mg/kg DON decreased final body weight, percentage of weight gain, specific growth rate and feed efficiency and induced body malformation. 0.922–1.515 mg/kg DON decreased intestinal somatic index.	(1) 0.027 mg/kg (control) (2) 0.318 mg/kg (3) 0.636 mg/kg (4) 0.922 mg/kg (5) 1.243 mg/kg (6) 1.515 mg/kg
Huang et al., 2019 [35]	*Ctenopharyngodon idella*	60 days	0.318–1.515 mg/kg DON decreased the glucose concentration in middle intestine. The effects on mRNA and protein expression suggest impaired immune function in intestines. 0.636–1.515 mg/kg DON increased enteritis morbidity after infection with *Aeromonas hydrophila* and decreased activities of lysozyme and acid phosphatase, as well as content of IgM, complement 3, and complement 4 in intestines.	(1) 0.027 mg/kg (control) (2) 0.318 mg/kg (3) 0.636 mg/kg (4) 0.922 mg/kg (5) 1.243 mg/kg (6) 1.515 mg/kg
Huang et al., 2020 [36]	*Ctenopharyngodon idella*	60 days	0.318–1.515 mg/kg DON affected biomarkers of oxidative damage and antioxidant status in the gill. The effects on mRNA and protein expression suggest tissue damage in the gill.	(1) 0.027 mg/kg (control) (2) 0.318 mg/kg (3) 0.636 mg/kg (4) 0.922 mg/kg (5) 1.243 mg/kg (6) 1.515 mg/kg
Tola et al., 2015 [66]	*Oreochromis niloticus × O. mossambicus*	56 days	Dose-dependent effect of co-exposure to DON and ZEN (Diet 1: 0.07/0.01 mg/kg, Diet 2: 0.31/0.09 mg/kg, Diet 3: 0.50/0.21 mg/kg, Diet 4: 0.92/0.37 mg/kg, Diet 5: 1.15/0.98 mg/kg): decrease in weight gain, thermal-unit growth coefficient, feed intake and feed efficiency; increase in mortality.	Co-contamination DON/ZEN: (1) 0.07/0.01 mg/kg (control) (2) 0.31/0.09 mg/kg (3) 0.50/0.21 mg/kg (4) 0.92/0.37 mg/kg (5) 1.15/0.98 mg/kg
Fornari et al., 2023 [67]	*Oreochromis niloticus*	71 days	1.6 mg/kg DON + 0.347 mg/kg ZEN decreased biomass gain and survival, caused histopathological changes in liver and increased hepatosomatic index.	(1) 0.2 mg/kg DON + 0.053 mg/kg ZEN (control) (2) 1.6 mg/kg DON + 0.347 mg/kg ZEN (either diet additionally contained 1.0–1.2 mg/kg fumonisins)
Trigo-Stockli et al., 2000 [38]	*Litopenaeus vannamei*	112 days	0.2–1 mg/kg DON decreased growth rate. 0.5–1 mg/kg DON decreased body weight.	(1) Uncontaminated control (DON not detected) (2) 0.2 mg/kg (3) 0.5 mg/kg (4) 1.0 mg/kg
Xie et al., 2018 [68]	*Litopenaeus vannamei*	35 days	0.362–0.958 mg/kg DON caused histopathological changes in hepatopancreas. 0.514–0.958 mg/kg DON caused histopathological changes in intestines. 0.958 mg/kg DON decreased weight gain and increased superoxide dismutase activity in hepatopancreas. Non-dose-dependent effect on glutathione peroxidase activity in hepatopancreas. Effects on mRNA expression of oxidative stress of genes related to immunity and oxidative stress defense.	(1) 0.171 mg/kg (control) (2) 0.362 mg/kg (3) 0.514 mg/kg (4) 0.958 mg/kg

^a^ Abbreviations: deoxynivalenol—DON; zearalenone—ZEN. ^b^ Concentrations are reported as fed unless stated otherwise.

**Table 4:** Adverse effects of dietary zearalenone concentrations at or below the EU guidance value (2 mg/kg) in fish and shrimps.

Publication	Species	Trial Duration	Effects of Concentrations at or Below Guidance Values ^a^	Dosages Tested (in mg ZEN/kg Feed) ^a,b^
Ghafarifarsani et al., 2021 [39]	*Oncorhynchus mykiss*	60 days	0.300–0.600 mg/kg ZEN decreased final body weight, weight gain, and specific growth rate and increased feed conversion ratio. Effects on digestive enzymes, immunological parameters in serum and intestines, oxidative stress biomarkers in serum and intestines, as well as intestinal histopathology were observed.	(1) 0.036 mg/kg (control) (2) 0.300 mg/kg (3) 0.600 mg/kg
Woźny et al., 2015 [40]	*Oncorhynchus mykiss*	71 days	1.81 mg/kg ZEN caused mild histopathological changes in the liver and advanced ovarian development.	(1) Uncontaminated control (ZEN not detected) (2) 1.81 mg/kg
Woźny et al., 2019 [41]	*Oncorhynchus mykiss*	672 days	2 mg/kg ZEN increased growth, decreased feed conversion ratio, caused inflammation of the trunk kidney, affected hematological parameters (e.g., decrease in B lymphocyte concentration) and cytokine expression in different organs.	(1) ≤0.099 mg/kg (mean: 0.0123 mg/kg; control) (2) ~2 mg/kg (1.876–2.132 mg/kg)
Woźny et al., 2020 [69]	*Oncorhynchus mykiss*	672 days	2 mg/kg ZEN increased mortality of offspring, caused morphological anomalies in gonads, and increased vitellogenin concentration in plasma of male fish.	(1) ≤0.099 mg/kg (mean: 0.0123 mg/kg; control) (2) ~2 mg/kg (1.876–2.132 mg/kg)
Ryerse et al., 2015 [58]	*Oncorhynchus mykiss*	28 days	3.3 mg/kg DON + 0.5 mg/kg ZEN significantly reduced feed intake and weight gain.	(1) <0.1 mg/kg (control) (2) 3.3 mg/kg DON + 0.5 mg/kg ZEN
Ryerse et al., 2016 [59]	*Oncorhynchus mykiss*	49 days	Dose-dependent effect of DON + ZEN (4.1 mg/kg DON + 0.5 mg/kg ZEN; 5.9 mg/kg DON + 0.6 mg/kg ZEN): reduction in feed intake and reduction in mortality following a *Flavobacterium psychrophilum* challenge.	(1) <0.5 mg/kg (control) (2) 4.1 mg/kg DON + 0.5 mg/kg ZEN (3) 5.9 mg/kg DON + 0.6 mg/kg ZEN
Pietsch and Junge, 2016 [70]	*Cyprinus carpio* L.	28 days	0.332–0.797 mg/kg ZEN affected lipid peroxidation in spleen, liver, head kidney, and gills, as well as enzymatic activity in spleen, liver, serum, and muscle. 0.621–0.797 mg/kg ZEN increased oxygen consumption.	(1) Uncontaminated control (ZEN not detected) (2) 0.332 mg/kg (3) 0.621 mg/kg (4) 0.797 mg/kg
Pietsch, et al., 2015 [71]	*Cyprinus carpio* L.	28 days	0.332–0.797 mg/kg ZEN had varying non-dose-dependent effects on immune function of leukocytes isolated from head kidney and trunk kidney.	(1) Uncontaminated control (ZEN not detected) (2) 0.332 mg/kg (3) 0.621 mg/kg (4) 0.797 mg/kg
Pietsch, et al., 2015 [72]	*Cyprinus carpio* L.	28 days	0.332–0.797 mg/kg ZEN increased micronucleus formation in erythrocytes. 0.621–0.797 mg/kg ZEN decreased monocyte and granulocyte counts.	(1) Uncontaminated control (ZEN not detected) (2) 0.332 mg/kg (3) 0.621 mg/kg (4) 0.797 mg/kg
Zhang, et al., 2023 [73]	*Ctenopharyngodon idella*	70 days	0.535–2.507 mg/kg ZEN affected immune parameters in the gill, as well as mRNA expression of tight junction-related, apoptosis-related, antioxidant-related, and immunity-related genes. 1.041–2.507 mg/kg ZEN caused histopathological changes in the gill, altered oxidative stress biomarkers in the gill, increased gill rot morbidity upon *Flavobacterium columnare* challenge, and affected protein expression of TOR phosphorylation, NF-κB p65, and Nrf2 in the gill.	(1) Uncontaminated control (ZEN not detected) (2) 0.535 mg/kg (3) 1.041 mg/kg (4) 1.548 mg/kg (5) 2.002 mg/kg (6) 2.507 mg/kg
Zhang, et al., 2021 [74]	*Ctenopharyngodon idella*	70 days	0.535–2.507 mg/kg ZEN decreased TOR phosphorylation in the hindgut. 1.041–2.507 mg/kg ZEN increased enteritis morbidity upon *Aeromonas hydrophila* challenge, affected immune parameters in the intestines, decreased protein expression of T-TOR and TOR phosphorylation in different gut segments, and increased NF-κB p65 protein expression in foregut and hindgut.	(1) Uncontaminated control (ZEN not detected) (2) 0.535 mg/kg (3) 1.041 mg/kg (4) 1.548 mg/kg (5) 2.002 mg/kg (6) 2.507 mg/kg
Wang et al., 2019 [42]	*Ctenopharyngodon idella*	70 days	0.535–2.507 mg/kg ZEN decreased Nrf2 protein level in the proximal intestine. 1.041–2.507 mg/kg ZEN decreased final body weight, weight gain, specific growth rate, feed intake, feed efficiency, intestine length, intestine weight, and intestinal somatic index, induced body line irregularities, caudal fin deformity, and intestinal swelling, and altered oxidative stress biomarkers in intestines.	(1) Uncontaminated control (ZEN not detected) (2) 0.535 mg/kg (3) 1.041 mg/kg (4) 1.548 mg/kg (5) 2.002 mg/kg (6) 2.507 mg/kg
Tola et al., 2015 [66]	*Oreochromis niloticus × O. mossambicus*	56 days	Dose-dependent effect of co-exposure to DON and ZEN (Diet 1: 0.07/0.01 mg/kg, Diet 2: 0.31/0.09 mg/kg, Diet 3: 0.50/0.21 mg/kg, Diet 4: 0.92/0.37 mg/kg, Diet 5: 1.15/0.98 mg/kg): decrease in weight gain, thermal-unit growth coefficient, feed intake and feed efficiency; increase in mortality.	Co-contamination DON/ZEN: (1) 0.07/0.01 mg/kg (control) (2) 0.31/0.09 mg/kg (3) 0.50/0.21 mg/kg (4) 0.92/0.37 mg/kg (5) 1.15/0.98 mg/kg
Abdel-Tawwab et al., 2020 [43]	*Dicentrarchus labrax*	28 days	0.725 mg/kg ZEN decreased final weight, weight gain, specific growth rate, and feed intake, decreased survival upon *Vibrio alginolyticus* challenge, and altered hematological and biochemical blood parameters.	(1) Uncontaminated control (ZEN not detected) (2) 0.725 mg/kg diet

^a^ Abbreviations: deoxynivalenol—DON; zearalenone—ZEN. ^b^ Concentrations are reported as fed unless stated otherwise.

**Table 5:** Adverse effects of dietary fumonisin concentrations at or below the EU guidance value (10 mg/kg) in fish and shrimps.

Publication	Species	Trial Duration	Effects of Concentrations at or Below Guidance Values ^a^	Dosages Tested (in mg/kg Feed) ^a,b^
Koletsi et al., 2023 [48]	*Oncorhynchus mykiss*	56 days (42 days restrictive feeding + 14 days ad libitum feeding)	Restrictive feeding: 8.798 mg/kg FB_1_ + FB_2_ inhibited growth, reduced final body weight and feed conversion and affected hepatosomatic index. Ad libitum feeding: 8.798 mg/kg FB_1_ + FB_2_ affected hepatosomatic index. 8.798 mg/kg FB_1_ + FB_2_ induced histopathological changes in liver and intestines.	(1) Uncontaminated control (FB_1_ + FB_2_ not detected) (2) 8.798 mg/kg FB_1_ + FB_2_ in dry matter (diet additionally contained 0.485 mg/kg fumonisin B_3_ and 2.696 mg/kg fusaric acid)
Petrinec et al., 2004 [75]	*Cyprinus carpio* L.	42 days	10 mg/kg FB_1_ caused pathological alterations in liver, endocrine, and exocrine pancreas, kidney, heart, and brain.	(1) Uncontaminated control (FB_1_ concentration not reported) (2) 10 mg/kg FB_1_ in dry matter
Kovacic et al., 2009 [76]	*Cyprinus carpio* L.	42 days	10 mg/kg FB_1_ decreased weight gain and caused neuronal apoptosis in the brain.	(1) Uncontaminated control (FB_1_ concentration not reported) (2) 10 mg/kg FB_1_ in dry matter
Baldissera et al., 2020 [44]	*Rhamdia quelen*	30 days	6.2 mg/kg FB_1_ decreased splenic nucleoside triphosphate diphosphohydrolase activity, glutathione peroxidase activity, and superoxide dismutase activity, and increased splenic adenosine deaminase activity as well as NOx levels. Total blood thrombocyte counts were decreased. Histopathological changes were observed in the spleen.	(1) Uncontaminated control (FB_1_ concentration not reported) (2) 6.2 mg/kg FB_1_
Baldissera et al., 2020 [45]	*Rhamdia quelen*	30 days	6.2 mg/kg FB_1_ decreased final mean weight, growth rate and weight gain and increased feed conversion ratio. Brain reactive oxygen species, lipid peroxidation and protein carbonylation were increased. Brain antioxidant capacity against peroxyl radical levels, as well as catalase, glutathione peroxidase and glutathione S-transferase activities were decreased. Histopathological changes were observed in the brain.	(1) Uncontaminated control (FB_1_ concentration not reported) (2) 6.2 mg/kg FB_1_
Gbore et al., 2010 [77]	*Clarias gariepinus*	42 days	5–15 mg/kg FB_1_ decreased weight gain and altered hematological and serum biochemical parameters. EFSA CONTAM Panel [78] concluded that this study could not be used to establish a safe limit due to limitations in experimental design and reporting. Therefore, the study was not considered for risk assessment in the present study either.	(1) Uncontaminated control (FB_1_ concentration not reported) (2) 5 mg/kg FB_1_ (3) 10 mg/kg FB_1_ (4) 15 mg/kg FB_1_
García-Morales et al., 2015 [46]	*Litopenaeus vannamei*	30 days	0.2–2 mg/kg FB_1_ decreased soluble muscle protein concentration and caused changes in myosin thermodynamic properties. 0.6–2 mg/kg FB_1_ decreased weight gain.	(1) Uncontaminated control (FB_1_ concentration not reported) (2) 0.2 mg/kg FB_1_ (3) 0.6 mg/kg FB_1_ (4) 2 mg/kg FB_1_
Mexia-Salazar et al., 2008 [79]	*Litopenaeus vannamei*	16 or 18 days	0.5–1.0 mg/kg FB_1_ caused histopathological changes in hepatopancreas, reduced prophenoloxidase activity, phenoloxidase activity, hemocyte count and superoxide anion production, and caused changes in electrophoretic pattern and thermodynamic properties of myosin.	(1) Uncontaminated control (FB_1_ not detected) (2) 0.5 mg/kg FB_1_ (3) 0.75 mg/kg FB_1_ (4) 1 mg/kg FB_1_
Kracizy et al., 2021 [80]	*Litopenaeus vannamei*	42 days	1.7153 mg/kg FB_1_ + FB_2_ decreased final weight, biomass gain, and specific growth rate and caused histopathological changes in hepatopancreas.	(1) Uncontaminated control (FB_1_ + FB_2_ not detected) (2) 1.7153 mg/kg FB_1_ + FB_2_

^a^ Abbreviations: fumonisin B_1_—FB_1_; fumonisin B_2_—FB_2_. ^b^ Concentrations are reported as fed unless stated otherwise.

**Table 6:** Fractions of compound feed samples exceeding mycotoxin concentrations that caused adverse effects in fish or shrimps in published studies.

	Deoxynivalenol	Zearalenone	Fumonisins B_1_ + B_2_ ^a^
Fish feed	9.3% ^b^	2.7% ^c^	0.4% ^d^
Shrimp feed	8.2% ^e^	- ^f^	4.9% ^g^

^a^ Sum of fumonisins B^1^ and B^2^. ^b^ Percentage of samples exceeding 0.318 mg/kg feed, i.e., the concentration that caused adverse effects in studies by Huang et al. [35,36,37]. ^c^ Percentage of samples exceeding 0.3 mg/kg feed, i.e., the concentration that caused adverse effects in a study by Ghafarifarsani et al. [39]. ^d^ Percentage of samples exceeding 6.2 mg/kg feed, i.e., the concentration that caused adverse effects in studies by Baldissera et al. [44,45]. ^e^ Percentage of samples exceeding 0.2 mg/kg feed, i.e., the concentration that caused adverse effects in a study by Trigo-Stockli et al. [38]. ^f^ No percentage was calculated, as no published studies were found that report adverse effects of dietary zearalenone concentrations below the EU guidance value in shrimps. ^g^ Percentage of samples exceeding 0.2 mg/kg feed, i.e., the concentration that caused adverse effects in a study by García-Morales et al. [46].

### 2.3. Mycotoxin Occurrence in Feed Raw Materials and Comparison to EU Limits

In maize, *Fusarium* mycotoxins FB_1_ + FB_2_, DON, and ZEN were prevalent (detected in 73.3%, 70.4%, and 53.7% of samples, respectively; Table 7). FB_1_ + FB_2_ and DON were detected in markedly higher concentrations compared to non-maize-based raw materials (see below), with median values of 422.0 µg/kg and 319.6 µg/kg and 90th percentile values of 3193.9 µg/kg and 1622.7 µg/kg, respectively. AFB_1_ was detected in 14.4% of the samples and 5.0% of the samples exceeded the maximum level for AFB_1_ stipulated for maize used as feed material (i.e., 20 µg/kg; Table 2). The most prevalent emerging mycotoxins in maize were moniliformin (detected in 62.0% of the samples) and beauvericin (detected in 54.2% of the samples; Table 7). In total, 98.3% of the maize samples were found to be contaminated with at least one mycotoxin or fungal metabolite, and 95.6% of the samples were contaminated with at least one regulated mycotoxin (Figure 1).

The maize product DDGS showed a similar mycotoxin occurrence pattern as maize (Table 7). However, both the prevalence and concentrations of *Fusarium* mycotoxins were markedly higher in DDGS than in maize (Table 7) and 100% of the samples were contaminated with at least one regulated mycotoxin (Figure 1). FB_1_ + FB_2_, DON, and ZEN were detected in 97.3%, 92.6%, and 96.0% of the samples at relatively high median concentrations of 663.8 µg/kg, 2114.4 µg/kg, and 169.9 µg/kg, respectively. In addition to DON, its metabolites DON-3-glucoside and 15-Acetyl-DON were detected in a high fraction of the samples (83.2% and 71.8%, respectively). Furthermore, ZEN metabolites β-zearalenol and α-zearalenol were detected in 71.1% and 45.6% of the samples, respectively. Emerging *Fusarium* mycotoxins moniliformin, beauvericin, and enniatin B were detected in 87.2%, 87.2%, and 51.0% of the samples, respectively. In addition, the emerging mycotoxins alternariol and sterigmatocystin were detected in 55.7% and 44.3% of the samples, respectively

In total, 92.9% of the wheat samples were contaminated with at least one mycotoxin or fungal metabolite, and 76.2% of the samples were contaminated with at least one regulated mycotoxin (Figure 1). In wheat, trichothecenes were prevalent (Table 8). DON was detected in 68.9% of the samples and reached relatively high concentrations (Table 8), with 1.3% of the samples exceeding the EU guidance value (Table 2). Furthermore, the DON metabolite DON-3-glucoside was detected in 50.3% of the samples and nivalenol was detected in 32.5% of the samples. In addition, ZEN was detected in 29.1% of the samples. Several of the enniatins were prevalent (e.g., enniatin B1 was detected in 71.8% of the samples), and moniliformin and beauvericin were detected in 40.5% and 38.2% of the samples, respectively. Ergot alkaloids showed a low prevalence in all raw materials including wheat (Table 7 and Table 8). However, the small fraction of wheat samples that did contain ergot alkaloids was contaminated at markedly higher levels compared to the other raw materials (e.g., a relatively high 90th percentile value of 270.6 µg/kg for ergocristine; Table 8).

In soybeans, regulated mycotoxins were less prevalent compared to other feed raw materials analyzed (detected in 53.3% of the samples; Figure 1). Still, 87.4% of the samples were contaminated with at least one mycotoxin or fungal metabolite. The most prevalent regulated mycotoxin was ZEN, which was detected in 32.7% of the samples (Table 8). In addition, emerging mycotoxins beauvericin and enniatin B were detected in 62.4% and 45.3% of the samples, respectively.

In rice, AFB_1_ was more prevalent than in most other raw materials (24.6% of positive samples; Table 8) and 4.6% of the samples exceeded the EU maximum level (Table 2). Furthermore, rice showed a high prevalence of FB_1_ + FB_2_ (detected in 81.5% of the samples) and ZEN (detected in 61.5% of the samples). The most prevalent emerging mycotoxins were sterigmatocystin and moniliformin, which were detected in 67.7% and 58.5% of the samples, respectively. In total, 95.4% of the rice samples were contaminated with at least one mycotoxin or fungal metabolite, and 93.8% of the samples were contaminated with at least one regulated mycotoxin (Figure 1).

### 2.4. Mycotoxin Co-Occurrence

**Figure 3:**
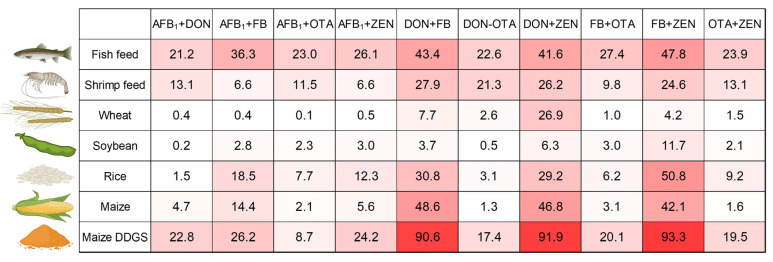
Frequency of binary mycotoxin combinations in compound feed and feed raw materials. The figure shows percentages of samples co-contaminated with any combination of two mycotoxins that are subjected to an EU maximum level (i.e., aflatoxin B_1_) or guidance value (i.e., deoxynivalenol, zearalenone, ochratoxin A, and the sum of fumonisins B_1_ and B_2_). The intensity of red in the heatmap illustrates the degree of co-contamination (more intense red indicates a higher percentage of co-contaminated samples). Abbreviations: aflatoxin B_1_—AFB_1_; deoxynivalenol—DON; sum of fumonisins B_1_ and B_2_—FB; ochratoxin A—OTA; zearalenone—ZEN. Illustrations of animals and feed raw materials were created in Biorender.

To analyze the co-occurrence of mycotoxins in each type of compound feed and feed raw material, we calculated (i) the fraction of samples co-contaminated with at least 5 or 10 of the 51 mycotoxins and fungal metabolites analyzed, (Figure 1), (ii) the fractions of samples co-contaminated with a least 2 or 3 of the regulated mycotoxins analyzed (Figure 1), and (iii) the fractions of samples co-contaminated with each combination of 2 regulated mycotoxins (Figure 3).

In the case of raw materials, the highest fractions of co-contaminated samples were observed for DDGS (Figure 1). In total, 99.3% and 86.6% of DDGS samples contained ≥ 5 and ≥10 mycotoxins/fungal metabolites, respectively, and 97.3% and 94.6% of DDGS samples contained ≥ 2 and ≥3 regulated mycotoxins, respectively. In most of the other raw materials, mycotoxin co-contamination was common as well. In the case of maize, wheat, and rice, 76.2%, 64.6%, and 60.0% of samples were contaminated with ≥5 mycotoxins/fungal metabolites and 73.6%, 33.5%, and 67.7% of samples were contaminated with ≥2 regulated mycotoxins, respectively. In most raw materials, combinations of *Fusarium* mycotoxins were prevalent (e.g., 48.6%, 46.8%, and 42.1% of maize samples were co-contaminated with DON + fumonisins, DON + ZEN, and fumonisins + ZEN, respectively (Figure 3). In addition, in DDGS, co-contamination of *Fusarium* mycotoxins and AFB_1_ was observed in >20% of the samples (Figure 3).

In the case of compound feed destined for fish, higher fractions of co-contaminated samples were observed compared to each raw material, except for the heavily contaminated material DDGS (Figure 1). In total, 75.7% and 39.4% of fish feed samples were contaminated with ≥5 and ≥10 mycotoxins/fungal metabolites, respectively, and 75.7% and 54.0% of the samples were contaminated with ≥2 and ≥3 regulated mycotoxins, respectively. Furthermore, any binary combination of regulated mycotoxins was detected in >20% of the samples (Figure 3). The most prevalent combinations were fumonisins + ZEN (47.8%), DON + fumonisins (43.4%), DON + ZEN (41.6%), and AFB_1_ + fumonisins (36.3%). Co-contamination was less common in compound feed destined for shrimps (Figure 1). In the case of shrimp feed, 55.7% and 26.2% of samples contained ≥ 5 and ≥10 mycotoxins/fungal metabolites, respectively, and 55.7% and 31.1% of samples contained ≥ 2 and ≥3 regulated mycotoxins, respectively.

## 3. Discussion

### 3.1. Aflatoxin Contamination of Aquaculture Feed

In this survey we found the global prevalence of AFB_1_ in 226 fish feed samples to be 41.6% with a median value of 1.6 µg/kg and a relatively high 90th percentile value of 93.8 µg/kg. Most previously published smaller surveys analyzing aflatoxin occurrence in fish feeds also found a high prevalence of aflatoxins. For example, a survey of fish feed from Argentina detected aflatoxins in 50% of the samples (n = 28) at a median concentration of 2.82 µg/kg [81] and a survey of fish feed samples from Brazil (n = 60) detected AFB_1_ in 55% of the samples (concentrations were not quantified) [82]. Of 37 fish and shrimp feed samples collected from Europe and Asia, 59% were contaminated with aflatoxins at an average concentration of 49 µg/kg [33]. In another survey of European aquaculture feed comprising 44 samples, only 5% of samples contained AFB_1_ at a mean concentration of 2 µg/kg [34]. A survey of fish feed samples from Kenya (n = 81) showed high contamination levels, as aflatoxins were detected in 84% of the samples at a median concentration of 3.6 µg/kg and 18.5% exceeded the maximum level of 10 µg/kg [83]. A survey of fish feed from Uganda found 63% of samples collected from farms and 48% of samples from factories to be contaminated with AFB_1_, at mean concentrations of 70–374 µg/kg for each farm and 0–211 µg/kg for each factory [84]. For AFB_1_ occurrence in shrimp feed, there is very little data published. In total, four out of seven analyzed shrimp feed samples from China [85] and one out of four analyzed shrimp feed samples from Southeast Asia [86] were contaminated with AFB_1_ concentrations exceeding the EU maximum level. The results of these surveys clearly indicate a high risk of aflatoxin contamination of aquaculture feedstuffs globally.

A subset of the fish feed samples analyzed in this survey showed high contamination levels, with 7.1% exceeding the maximum level for AFB_1_ that is in effect in the European Union (i.e., 10 µg/kg). Adverse effects of dietary AFB_1_ concentrations above the maximum level are well documented in different fish species. For example, in Tra catfish (*Pangasius hypophthalmus*) 50–250 µg AFB_1_/kg diet reduced weight gain and specific growth rate, increased liver enzyme activity in serum, and increased mortality following a challenge with *Edwardsiella ictaluri* [87]. Dietary exposure of gibel carp (*Carassius auratus gibelio*) to 20 µg/kg AFB_1_ decreased gonadosomatic index and absolute and relative brood amount, as well as oocyte diameter [88]. In rainbow trout (*Oncorhynchus mykiss*), a diet contaminated with 80 µg AFB_1_/kg caused histopathological changes in the liver [89]. Furthermore, rainbow trout have famously been used as a model system for the investigation of the carcinogenic effect of AFB_1_ since the 1950s [90] and dietary AFB_1_ concentrations as low as 0.5 µg/kg have been found to be carcinogenic [91,92]. Noteworthily, in a recent study, a dietary AFB_1_ concentration of 7 µg/kg—i.e., below the EU maximum level of 10 µg/kg—fed for 8 weeks was found to reduce the final body weight of hybrid grouper (*Epinephelus fuscoguttatus* ♀ × *Epinephelus lanceolatus* ♂) [93]. In our survey, 9.3% of fish feed samples exceeded 7 µg/kg AFB_1_. According to these published studies, low dietary AFB_1_ concentrations can negatively affect aquaculture fish, and the EU maximum level may not be fully protective for fish health and growth performance.

With 16.4% positive samples, AFB_1_ was less prevalent in shrimp feed than in fish feed (Table 1). Furthermore, in positive samples, contamination levels were a bit lower, and all but one sample complied with the EU maximum level. Lower contamination levels in shrimp feed compared to fish feed could be due to investment in high quality feed for high value shrimp species. According to published studies, Pacific white shrimp (*Litopenaeus vannamei*) are sensitive to aflatoxins. A dietary aflatoxin concentration of 75 µg/kg fed for 42 days decreased weight gain, feed intake, and nitrogen retention efficiency [94]. Furthermore, a dietary AFB_1_ level of 50 µg/kg fed for one week caused lesions in hepatopancreas and antennal gland tissues [95]. In black tiger shrimp (*Penaeus monodon*) even lower dietary AFB_1_ concentrations of 5–20 µg/kg decreased body weight, increased mortality, and caused histopathological changes in the hepatopancreas [96]. Therefore, AFB_1_ contamination in shrimp feed should be minimized.

### 3.2. Deoxynivalenol, Zearalenone, and Fumonisin Contamination of Aquaculture Feed

The *Fusarium* mycotoxins DON, ZEN, and FB_1_ + FB_2_ were prevalent in both fish and shrimp feed (Table 1). Previously published surveys comprising smaller sample sizes confirm the high prevalence of DON, ZEN, and fumonisins in aquaculture feed globally. For example, DON, ZEN, and FB_1_ + FB_2_ were detected in 68%, 59%, and 51% of fish and shrimp feed samples (n = 41) collected in Europe and Asia [33]. Furthermore, in samples of fish feeds and ingredients from Kenya (n = 78), DON, ZEN, and FB_1_ reached a prevalence of 76%, 40%, and 54%, respectively [97]. Furthermore, 90% of fish feed samples from Brazil (n = 60) were contaminated with fumonisins, and 50% and 4% of fish feed samples from Argentina (n = 28) were contaminated with ZEN and DON, respectively [81].

The vast majority of samples analyzed in this survey complied with EU guidance values (Table 2). Regulation of mycotoxin concentrations in animal feed is more extensive in the EU than in most other countries and regions. Furthermore, the risk of mycotoxin contamination in feed to animals is frequently re-evaluated by EFSA panels based on the most recent information from scientific literature and feed surveys. For example, based on recent literature, reference points for fumonisins and DON for poultry feed have been lowered by EFSA panels in 2022 [98] and 2023 [99], respectively. In the case of aquatic animals, an EFSA panel identified a no-observed-adverse-effect level of 0.6 mg DON/kg feed for carps, rainbow trout, and salmon in 2017, which is markedly lower than the EU guidance value of 5 mg/kg [100]. In this study, we aimed to perform a risk assessment based on the most recent literature and feed survey data. We found that significant fractions of compound feed samples were contaminated with concentrations shown to cause adverse effects in published studies (Table 6). These results suggest that certain fractions of feed samples used in aquaculture are a cause for concern for fish and shrimp health and growth despite compliance with EU guidance values.

### 3.3. Emerging Mycotoxins in Aquaculture Feed

In addition to the regulated mycotoxins, several emerging mycotoxins were frequently detected in fish and shrimp feed, including enniatins, beauvericin, alternariol, moniliformin, sterigmatocystin, and mycophenolic acid (Table 1). Two recent studies investigated the effects of beauvericin and enniatin B in Atlantic salmon (*Salmo salar*). Based on the results of a dose–response study, Berntssen and coworkers [101] concluded that the safe limit for effects on growth performance is 20–50 µg/kg feed for enniatin B and 912–1504 µg/kg feed for beauvericin. In addition, dietary beauvericin levels of ≥300 µg/kg affected hematology and vitamin E levels in the liver [101]. In a study by Soderstrom and coworkers [102], a single oral dose of feed contaminated with 50 or 500 μg/kg beauvericin, or 50 or 500 μg/kg enniatin B affected global gene transcription in the liver and intestines of Atlantic salmon. Results of transcriptome analysis suggested that beauvericin induced pathways linked to hepatic hematological disruption, while enniatin B induced pathways linked to intestinal inflammation. According to the results of these studies, concentrations detected in a subset of fish feed samples in this survey (Table 1) could be a cause for concern, both in the case of enniatins (90th percentile of positive samples for enniatin B and B_1_ was 46 µg/kg and 26 µg/kg, respectively) and beauvericin (90th percentile of positive samples: 66 µg/kg).

High dietary moniliformin concentrations far in excess of concentrations detected in this survey (Table 1) caused adverse effects in fish. Tuan and coworkers [103] reported that 70–150 mg/kg moniliformin (but not ≤ 40 mg/kg) fed to Nile tilapia (*Oreochromis niloticus*) for 8 weeks decreased weight gain and hematocrit levels in serum and increased the feed conversion ratio. In channel catfish (*Ictalurus punctatus*), 20–120 mg moniliformin/kg diet fed for 10 weeks decreased weight gain and feed consumption [104]. Furthermore, in a zebrafish model study, exposure of larvae to 900–1800 µg/L moniliformin in water for 3 days decreased survival, and exposure to 450 µg/L for 20 days decreased the standard length of fish [105]. The effects of chronic exposure to lower dietary moniliformin concentrations in the range of concentrations detected in this survey (Table 1) have not been investigated yet and should be clarified in future studies.

Mycophenolic acid has negligible toxic effects [25]. Therefore, its detection in fish feed is likely no cause for concern. In contrast, adverse effects in fish have been reported for sterigmatocystin. Sterigmatocystin is a precursor in aflatoxin biosynthesis and exerts similar effects as AFB_1_ albeit with lower acute toxicity [25]. In common carp (*Cyprinus carpio L.*), dietary sterigmatocystin concentrations of ≥1 mg/kg caused oxidative stress in the liver [106,107] and increased the mRNA expression of DNA repair genes in the hepatopancreas [108]. Furthermore, in an earlier study, dietary sterigmatocystin concentrations of 10–1250 µg/kg gradually decreased body weight gain, increased mortality, and increased liver enzyme activities in serum indicative of liver damage [109]. In the same study, in catfish (*Clarias gariepinus*), a dietary sterigmatocystin concentration of 250 µg/kg added to diets containing different crude protein levels consistently caused an increase in mortality and a decrease in body weight gain and body length [109]. While these studies indicate adverse effects of dietary sterigmatocystin in fish, additional studies should be performed to assess the risk of lower concentrations detected in fish feed in this survey (Table 1).

A plethora of ergot alkaloids was detected in fish and shrimp feed (Table 1). Published studies on the effects of ergot alkaloids in fish and shrimps are lacking and should be performed in the future to assess the risk of these compounds to aquaculture species.

To the best of our knowledge, no studies have been published on the effects of enniatins and beauvericin in aquaculture shrimp species, or on alternariol in fish species. The effects of these compounds in shrimps and fish should be investigated given their frequent occurrence in aquaculture feed (Table 1).

### 3.4. Mycotoxin Occurrence in Feed Raw Materials

Each feed raw material analyzed exhibited a distinct mycotoxin occurrence pattern (Table 7 and Table 8) consistent with the well-known susceptibility of the respective crop plants to certain fungal pathogens. In maize, fumonisins, DON, and ZEN were prevalent and detected at high levels (Table 7), consistent with the susceptibility of maize plants to infestation by fumonisin producer *Fusarium verticillioides* [110] and DON and ZEN producing *Fusarium* species such as *Fusarium graminearum* [111]. A subset of both maize and rice samples was contaminated with high levels of AFB_1_ (Table 7 and Table 8), with 5.0% and 4.6% of maize and rice samples, respectively, exceeding the EU maximum level stipulated for feed raw materials (i.e., 20 µg/kg; Table 2). These results reflect the susceptibility of maize and rice kernels to infestation with aflatoxin-producing *Aspergillus* species both during plant growth and during storage at higher temperatures prevailing in tropical and subtropical regions [111,112,113]. Wheat is susceptible to infestation with *Fusarium graminearum* and other *Fusarium* species that produce DON and ZEN [114,115]. Accordingly, these mycotoxins were prevalent in wheat in this study (Table 8). Similarly to what was observed for the regulated mycotoxins, pronounced differences in the occurrence of emerging mycotoxins between different raw materials were detected. For example, particularly high fractions of positive samples and high concentrations were observed for enniatins in wheat (Table 8), moniliformin in maize (Table 7), and sterigmatocystin—a toxic aflatoxin precursor—in rice (Table 8).

Of the raw materials analyzed in this study, maize DDGS stand out due to particularly high percentages of positive samples and high detected concentrations for many regulated and emerging mycotoxins (Table 7). High mycotoxin contamination levels in DDGS are a result of their production process. DGGS are a cereal by-product of the bioethanol distillation process. If mycotoxin-contaminated grain is used as the starting material, mycotoxins accumulate in DDGS. For example, DON, ZEN, and fumonisins are enriched in DDGS to ~3 times the concentration present in the starting material [116,117,118,119]. In addition, DDGS frequently contain other contaminants such as antibiotics [120]. DDGS are nutrient-rich materials that are well suited as feed ingredients. However, concentrations of mycotoxins and other contaminants in DDGS must be closely monitored to ensure that use for animal nutrition is safe.

Raw materials with high prevalence of DON such as maize, DDGS, and wheat were often co-contaminated with the masked mycotoxin DON-3-glucoside and the fungal DON metabolite 15-acetyl-DON (Table 7 and Table 8). During digestion in terrestrial animals, the glucoside group of DON-3-glucoside and the acetyl group of 15-acetyl-DON are removed by gut bacteria and DON is absorbed into systemic circulation [100]. Consequently, DON-3-glucoside and 15-acetyl-DON can add to the toxicity of DON. It is, therefore, advisable to screen for DON-3-glucoside and 15-acetyl-DON (also 3-acetyl-DON) in addition to DON.

When raw materials are selected for the production of compound feed, it is important to consider their mycotoxin occurrence profiles and resulting mycotoxin concentrations after mixing with other feed ingredients. Additionally, it is important to consider that regulatory limits may permit higher mycotoxin concentrations in feed raw materials than in compound feed. Higher EU maximum levels and guidance values have been stipulated for feed raw materials compared to compound feed in the case of several mycotoxins and materials (see EU limits listed in Table 2). For example, the EU maximum level stipulated for AFB_1_ in compound feed is 10 µg/kg, whereas feed raw materials are permitted to contain up to 20 µg/kg AFB_1_. Furthermore, the EU guidance value for FB_1_ + FB_2_ in compound feed is 10 mg/kg, whereas a guidance value of 60 mg/kg has been stipulated for maize and maize DDGS. When feed raw materials are mixed for feed production, mycotoxin concentrations in the resulting blend must conform with legal requirements for compound feed.

### 3.5. Co-Occurrence of Mycotoxins

Co-occurrence of mycotoxins was common in both feed raw materials and compound feed destined for fish and shrimps (Figure 1). Co-contamination of raw materials traces back to the infestation of crop plants and stored grains with multiple mycotoxigenic fungi, as well as the simultaneous production of multiple mycotoxins by the same fungal strain. When feed raw materials with different mycotoxin occurrence patterns are blended to produce compound feed, even more complex mixtures of mycotoxins are created. Indeed, we found that higher percentages of fish feed samples were co-contaminated compared to raw materials, with the exception of the highly contaminated DDGS (Figure 1). Furthermore, in contrast to the raw materials, in fish feed, any combination of two regulated mycotoxins was detected in >20% of the samples (Figure 3). In the case of shrimp feed, slightly lower percentages of samples were co-contaminated compared to fish feed (Figure 1), in agreement with a lower prevalence of individual mycotoxins (Table 1). In summary, aquaculture feed is frequently contaminated with mycotoxin mixtures and consequently, fish and shrimps are exposed to multiple mycotoxins on a regular basis.

The consequences of dietary exposure to mycotoxin mixtures are the combined effects of these mixtures in animals. In general, combined effects of mycotoxins can be additive, synergistic, or antagonistic, that is equal to, greater than, or lower than the summed effects of the individual mycotoxins [29,30]. The effects of mycotoxin mixtures in animals have received some attention in recent years, yet knowledge on this subject is still limited. Surveying mycotoxin occurrence enables the identification of the most prevalent mycotoxin mixtures and, thus, can help to determine where research efforts should be focused. The most prevalent mixtures of regulated mycotoxins in aquaculture feed were combinations of fumonisins, ZEN, and DON, as well as the combination of AFB_1_ and fumonisins (Figure 3). In terrestrial animals, both combinations of fumonisins, ZEN, and DON and the combination of AFB_1_ and fumonisins have been reported to cause any type of combined effect (additive, synergistic, or antagonistic) [29]. The type of combined effect may differ according to the investigated endpoint, the applied mycotoxin dose, the exposure route, the duration of exposure, and the investigated species, as well as the animal’s age, sex, and nutritional status [29]. Additive or synergistic effects of combinations of fumonisins, ZEN, and DON were reported for endpoints such as growth performance [121,122,123], parameters of immune function [124,125,126,127], liver health [127,128,129], and gut health [123,130] in terrestrial animals. Furthermore, AFB_1_ + fumonisins exerted synergistic effects on growth performance [131,132,133] and liver health [133,134,135] in terrestrial species. Unfortunately, there is very little data available on the combined effects of mycotoxins in aquatic animals. In a recent study, dietary co-exposure to DON and fumonisins caused additive effects on growth performance of rainbow trout [48]. Furthermore, dietary fumonisins promoted liver tumors initiated by the administration of AFB_1_ in rainbow trout [136]. According to this body of knowledge available mostly for terrestrial species, exposure to mycotoxin mixtures frequently detected in this survey can result in an increased risk of adverse effects compared to exposure to single mycotoxins. Future studies should address combined effects of frequently co-occurring mycotoxins in fish and shrimps to support risk assessment.

### 3.6. Potential Risk of Mycotoxin Carry over to Edible Products

Assessing the potential risk to humans from the consumption of fish and shrimp fed mycotoxin-contaminated diets is challenging and focuses on a possible transfer and retention of the mycotoxin (bioaccumulation) in the consumed product. The bioaccumulation of different mycotoxins in fish species was recently summarized by Gonçalves et al. [137] and Tolosa et al. [138]. Bioaccumulation can occur even with no apparent mycotoxin exposure symptoms in the animal [139]. The fish species, the consumed tissue, the mycotoxin, its metabolites, the exposure pattern, and other factors must be considered along with estimated tolerable intake values to draw conclusions on the risk for human consumers. Hence, making a dichotomic determination of ‘safe’ or ‘unsafe’ is better performed only on a case-by-case basis and should not be generalized. Concerning the mycotoxin concentrations detected in feedstuffs in this study, feeds that contained higher concentrations of AFB_1_ above the maximum level could be a cause for concern for carry-over negatively affecting human consumers [137]. Concerning other mycotoxins, it is likely that bioaccumulation and the risk to human consumers as an outcome will be low. Nevertheless, it is not possible to entirely exclude a risk to humans based only on these results.

## 4. Conclusions

With >90% of samples contaminated with at least one mycotoxin, mycotoxins were almost ubiquitously present in fish and shrimp feed. In total, 7.2% of fish feed samples were contaminated with unsafe concentrations of AFB_1_ exceeding the EU maximum level. Most fish and shrimp feed samples complied with EU guidance values for DON, ZEN, and FB_1_ + FB_2_. However, according to the results of published studies, guidance values stipulated for these mycotoxins may not be fully protective for aquatic species and significant fractions of analyzed feed samples exceeded concentrations shown to exert adverse effects. Therefore, based on the results of this global survey, AFB_1_, DON, ZEN, and FB_1_ + FB_2_ are a burden to the global aquaculture industry.

Several less investigated, emerging mycotoxins were prevalent in aquaculture feed, including enniatins, beauvericin, alternariol, and moniliformin. Enniatin and beauvericin concentrations detected in a subset of fish feed samples could be a concern for fish health according to results reported in recent publications. No data are currently available on the effects of alternariol and moniliformin in fish at concentrations commonly detected in feed, and data on the effects of frequently detected emerging mycotoxins in shrimps are completely lacking. The high prevalence of these compounds in our aquaculture feed survey highlights the necessity to close these data gaps.

Single mycotoxins, not mycotoxin mixtures, are usually the subject of risk assessment and regulation. However, according to this survey, co-contamination of aquaculture feed is common. Therefore, combined effects of mycotoxin mixtures in aquatic animals upon dietary exposure are to be expected and should receive more attention. According to data mainly available for terrestrial animals, prevalent mycotoxin mixtures identified in this survey can exert additive or synergistic effects resulting in an increased risk to animal health. For fish and shrimps, targeted studies are needed to investigate the combined effects of the most frequently detected mycotoxin mixtures and to identify safe levels of these mixtures.

## 5. Materials and Methods

### 5.1. Collection of Feed Samples

Data analyzed in this study were derived from the dsm-firmenich World Mycotoxin Survey (previously known as the BIOMIN World Mycotoxin Survey), a global survey program monitoring mycotoxin contamination in feed that has been introduced in 2004. Studies on data derived from this survey have been published previously, e.g., [31,32,140,141]. Within this framework, a dedicated aquaculture feed survey has been performed in 2018–2023. For this purpose, 226 samples of compound feed for fish were collected from South America (n = 17), Sub-Saharan Africa (n = 30), Asia (n = 83), Europe (n = 85), and the Middle East/North Africa (n = 11) from August 2018 to April 2023. Furthermore, 61 samples of compound feed for shrimps were collected from Asia (n = 37), Central America (n = 18), South America (n = 4), and Europe (n = 2) from September 2019 to April 2023. In addition to compound feed for aquaculture, raw materials commonly used in aquaculture feed were analyzed. In total, 3448 maize samples were collected from South America (n = 313), North America (n = 91), Central America (n = 27), Sub-Saharan Africa (n = 1818), Oceania (n = 16), Asia (n = 501), Europe (n = 578), Middle East/North Africa (n = 24), and unknown regions of origin (n = 80) from May 2018 to March 2023. In total, 149 maize dried distillers’ grains with solubles (DDGS) samples were collected from North America (n = 42), Sub-Saharan Africa (n = 15), Oceania (n = 3), Asia (n = 42), Europe (n = 30), the Middle East/North Africa (n = 1), and unknown regions of origin (n = 16) from June 2018 to February 2022. In total, 1578 wheat samples were collected from South America (n = 29), North America (n = 11), Central America (n = 3), Sub-Saharan Africa (n = 49), Oceania (n = 96), Asia (n = 30), Europe (n = 1141), the Middle East/North Africa (n = 2), and unknown regions of origin (n = 217) from January 2018 to March 2023. In total, 428 soybean samples were collected from South America (n = 47), North America (n = 30), Sub-Saharan Africa (n = 237), Oceania (n = 1), Asia (n = 21), Europe (n = 85), the Middle East/North Africa (n = 2), and unknown regions of origin (n = 5) from January 2018 to March 2023. In total, 65 rice samples were collected from Asia (n = 56), Europe (n = 3), and unknown regions of origin (n = 6) from September 2019 to February 2023. Analyzed compound feed samples were obtained from diverse sources (ingredients of each compound feed were not disclosed). Ingredients and inclusion rates are expected to vary between different feedstuffs. All sampled raw materials were intended for feed production, for either terrestrial or aquatic species.

Sample collection, milling of samples and homogenization of samples was performed as described by Kovalsky et al. [141]. To avoid accumulation of humidity, samples were stored in paper bags. All samples were immediately sent to the laboratory for mycotoxin analysis. The approximate moisture content was 9.0% for compound feed, 12.3% for maize, 11.3% for maize DDGS, 13.5% for wheat, 11.2% for soybean and 13.6% for rice. Mycotoxin concentrations are reported as fed.

### 5.2. Mycotoxin Analysis

Samples were analyzed either (i) by Romer Labs GmbH (Tulln, Austria) using the LC-MS/MS-based multi-mycotoxin analysis method Spectrum Top^®^ 50, or (ii) at the Department of Agrobiotechnology (IFA-Tulln) of the University of Natural Resources and Life Sciences Vienna (BOKU) in Tulln, Austria, using an LC-MS/MS-based multi-mycotoxin analysis method described by Sulyok et al. [142], which enables the accurate quantification of >500 secondary metabolites of plants, bacteria, and fungi including all relevant mycotoxins (introduced into the market as Spectrum 380^®^ by BIOMIN Holding GmbH, now part of dsm-firmenich, in cooperation with BOKU/IFA-Tulln in 2014). For our evaluation, all substances were considered that are detected by both applied analysis methods, namely 15-acetyl-DON, 3-acetyl-DON, AFB_1_, aflatoxin B_2_, aflatoxin G_1_, aflatoxin G_2_, agroclavine, alpha-zearalenol, alternariol, beauvericin, beta-zearalenol, DON, DON-3-glucoside, diacetoxyscirpenol, dihydrolysergol, elymoclavine, enniatin A, enniatin A_1_, enniatin B, enniatin B_1_, ergine, ergocornine, ergocristine, ergocristinine, ergocryptine, ergocryptinine, ergometrine, ergometrinine, ergosine, ergotamine, FB_1_, FB_2_, fumonisin B_3_, fusarenon X, gliotoxin, HT-2 toxin, moniliformin, monoacetoxyscirpenol, mycophenolic acid, neosolaniol, nivalenol, OTA, ochratoxin B, patulin, penicillic acid, roquefortine C, sterigmatocystin, T-2 tetraol, T-2 toxin, T-2 triol, and ZEN. Each analysis method is briefly summarized in the following paragraphs. For more details on the limits of detection and the limits of quantification, calibration curves, and recovery, we would like to refer the reader to the previous publications by Sulyok et al. [142,143] and Steiner et al. [144]. In addition, limits of detection and limits of quantification, as well as apparent recovery, as reported previously, are listed in Table 9.

For analysis at Romer Labs GmbH (Tulln, Austria), all samples were measured in an ISO 17025 [145] accredited laboratory (Analytical Service, Lab, Romer Labs Diagnostic GmbH, Romer Labs^®^). Validation and quality controls of the method are in accordance with ISO 17025 requirements. For sample preparation, samples and for each batch, a known quality control sample is homogenized, weighed in, and extracted with 70% organic solvent, following shaking for 60 min. Samples are then centrifuged and diluted prior to the injection. Each mycotoxin analyzed is measured against a standard curve (calibration curve) that is analyzed in each batch at the beginning and the end of the LC-MS run. Standards and isotopic labels standards (IST) are mainly purchased from Biopure™, Romer Labs^®^ (ISTs are not commercially available for all mycotoxins). Samples were measured on an Agilent 1260 Infinity series high performance liquid chromatography (HPLC) system (Agilent Technologies, Waldbronn, Germany) coupled to a Sciex QTRAP6500+ mass spectrometer (Sciex, Foster City, CA, USA; multiple reaction monitoring mode, negative and positive mode were used). The applied LC column was Phenomenex Gemini C18 (4.6, 0 × 150 mm; 5 µm). The LC flow rate was 0.9 mL/minute, the injection volume was 10 µL for samples and 1 µL for IST and the run time was 31 min. Mycotoxins eluted with a gradient starting at 100% mobile phase A to 0% mobile phase A (mobile phase A 90% aqueous; mobile phase B 97% organic). For data evaluation, default values were used for the integration of the peaks (e.g., smoothing). Concentrations of samples were calculated based on a linear standard curve. Matrix effects were corrected with IST where available. The limits of detection and limits of quantification were estimated as described in the Eurachem Guide [146]. Recoveries were calculated based on spiked samples. The calibration curve (working range and linearity) was evaluated according to DIN 38402.

For analysis at IFA Tulln (Department of Agrobiotechnology of the University of Natural Resources and Life Sciences Vienna, BOKU, in Tulln, Austria) according to Sulyok et al. [142], samples were homogenized using a Blixer 4 (Robot Coupe, Vincennes, France) and 5 g of homogenized material was extracted using 20 mL of acetonitrile/water/acetic acid 79:20:1, *v*/*v*/*v* and shaken for 90 min on a rotary shaker (GFL 3017, GFL; Burgwedel, Germany). A total of 500 µL of the supernatants was transferred into HPLC vials and diluted with 500 µL of acetonitrile/water/acetic acid 20:79:1, *v*/*v*/*v*. After appropriate mixing, 5 µL of the diluted extracts were injected into the LC–MS/MS system without further pre-treatment. Mycotoxin analysis was carried out using a 1290 Series HPLC System (Agilent, Waldbronn, Germany) coupled to a QTrap 5500 LC-MS/MS System (Applied Biosystems SCIEX, Framingham, MA, USA) equipped with Turbo Ion Spray electrospray ionization source. Chromatographic separation was performed at 25 °C running an acidified methanol/water gradient on a Gemini^®^ C18-column, 150 × 4.6 mm i.d., 5 μm particle size, equipped with a C18 4 × 3 mm i.d. security guard cartridge (Phenomenex, Torrance, CA, USA). ESI-MS/MS data were acquired in the scheduled multiple reaction monitoring mode both in positive and negative polarity in two separate chromatographic runs. The detection window width was 40 and 46 sec in the positive and negative ionization mode, respectively. The target cycle time was 1400 msec and the MS pause time was 3 msec. Compound-dependent MS/MS parameters and source of reference standards are listed in Sulyok et al. [142]. Confirmation of positive metabolite identification was carried out by the acquisition of two MS/MS signals per analyte in the time-scheduled multiple reaction-monitoring mode, which yielded 4.0 identification points according to the European Commission decision 2002/657. In addition, retention time and ion ratio had to agree to the related values of authentic standards within 0.03 min and 30% rel., respectively. Quantitation was based on external calibration using serial dilutions of a multi-analyte stock solution. Results were corrected for apparent recoveries determined in feed. The performance of the method is verified on a continuous basis by participation in a proficiency testing scheme (BIPEA, Genneviliers, France) with >96% of the 2200 results submitted so far exhibiting a z-score of −2 < z < 2. Limits of detection and limits of quantification were determined according to the Eurachem guide [146].

### 5.3. Comparison of Mycotoxin Concentrations to EU Regulatory Limits and Concentrations Reported to Cause Harmful Effects in the Literature

To assess the risk of mycotoxin occurrence in feed to fish and shrimp, the percentages of samples exceeding EU maximum levels (in the case of AFB_1_) or guidance values (in the case of DON, ZEN, OTA and FB_1_ + FB_2_) [22,23,24] were calculated. We used EU limits for this assessment, as European legislation on mycotoxin contamination in feed is extensive, whereas many other countries have stipulated limits only for some of these mycotoxins or lack regulation of mycotoxins in feed altogether.

To further characterize the risk of low contamination levels, a literature search was performed to identify published studies on the effects of DON, ZEN, and fumonisins at concentrations at or below the EU guidance values applicable for aquaculture feed (DON: 5 mg/kg; ZEN: 2 mg/kg; FB_1_ + FB_2_: 10 mg/kg). For this purpose, public literature databases Pubmed (https://pubmed.ncbi.nlm.nih.gov/) and Scopus (https://www.scopus.com/) were searched on 17 and 18 April 2024 using default search parameters and the following keywords: (1) for literature on the effects of ZEN, DON, and fumonisins in shrimps: shrimp AND (zearalenone OR deoxynivalenol OR fumonisin), (2) for literature on effects of ZEN, DON, and fumonisins in aquaculture fish: (trout OR salmon OR catfish OR tilapia OR carp OR “sea bass”) AND (zearalenone OR deoxynivalenol OR fumonisin). After removal of duplicates, as well as conference papers and papers written in languages other than English, relevant publications were identified by screening titles and abstracts. Publications were considered relevant if they reported the effects of dietary DON, ZEN, or fumonisin concentrations at or below guidance values in fish or shrimps with an experimental design that included one or more mycotoxin-treated groups and a control group that did not receive mycotoxins (or only low background concentrations of mycotoxins occurring naturally in the basal diet). Studies that administered mycotoxins via the diet but did not give mycotoxin concentrations in the diet were excluded. Furthermore, studies that only analyzed the effects of mycotoxins on mRNA expression were excluded.

## Figures and Tables

**Table 1:** Mycotoxin occurrence in compound feed destined for fish and shrimps.

	Compound Feed for Fish (n = 226)				Compound Feed for Shrimps (n = 61)			
Mycotoxin	Prevalence (% Positive Samples)	Median of Positives (µg/kg)	90th Percentile of Positives (µg/kg)	Maximum (µg/kg)	Prevalence (% Positive Samples)	Median of Positives (µg/kg)	90th Percentile of Positives (µg/kg)	Maximum (µg/kg)
**Regulated mycotoxins (EU maximum level or guidance value)**								
Aflatoxin B_1_	41.6	1.6	93.8	1774.3	16.4	1.0	7.0	15.5
Fumonisins B_1_ + B_2_^a^	68.6	138.8	693.8	13,567.1	41.0	24.9	245.7	415.7
Deoxynivalenol	58.0	124.3	471.4	1867.2	60.7	113.4	294.5	1025.6
Zearalenone	61.1	24.9	139.9	1045.4	37.7	12.5	67.4	91.9
Ochratoxin A	33.6	2.2	7.6	21.3	27.9	1.0	5.5	11.0
**Other aflatoxins**								
Aflatoxin B_2_	8.8	10.5	74.3	194.4	1.6	1.5	1.5	1.5
Aflatoxin G_1_	12.4	6.2	37.2	79.1	3.3	2.5	3.2	3.4
Aflatoxin G_2_	3.5	3.8	11.2	12.4	Not detected.			
**Fumonisins**								
Fumonisin B_1_	65.9	96.8	458.0	9074.7	27.9	15.0	258.8	295.7
Fumonisin B_2_	64.6	56.2	237.7	4492.4	34.4	15.0	97.3	120.0
Fumonisin B_3_	52.2	24.5	109.8	1353.2	14.8	19.5	40.7	51.3
**Other trichothecenes**								
Deoxynivalenol-3-glucoside	23.9	25.0	75.0	147.3	24.6	25.0	25.0	25.5
15-Acetyl-deoxynivalenol	0.9	75.0	75.0	75.0	Not detected.			
3-Acetyl-deoxynivalenol	0.4	7.7	7.7	7.7	Not detected.			
Nivalenol	4.0	66.8	93.6	143.0	Not detected.			
T-2 toxin	5.3	2.6	11.3	14.1	Not detected.			
HT-2 toxin	1.8	25.0	25.0	25.0	1.6	25.0	25.0	25.0
T-2 Tetraol	2.2	8.7	11.3	11.9	Not detected.			
Monoacetoxyscirpenol	1.8	3.9	12.0	15.0	Not detected.			
Diacetoxyscirpenol	1.8	5.0	9.4	11.3	Not detected.			
Neosolaniol	0.4	1.5	1.5	1.5	Not detected.			
Fusarenon-X	1.3	159.2	307.4	344.4	4.9	131.7	261.5	294.0
**Zearalenone metabolites**								
beta-Zearalenol	12.4	10.0	19.3	99.4	6.6	10.0	10.0	10.0
alpha-Zearalenol	2.2	10.0	10.0	10.0	Not detected.			
**Other ochratoxins**								
Ochratoxin B	8.0	1.5	8.1	8.9	6.6	3.7	5.9	6.0
**Emerging mycotoxins (less investigated fungal metabolites)**								
Enniatin B	46.9	6.6	45.9	120.2	42.6	8.0	27.8	49.7
Enniatin B1	54.0	6.0	25.6	70.1	37.7	7.8	15.3	23.9
Enniatin A	7.5	10.0	10.0	10.0	1.6	1.5	1.5	1.5
Enniatin A1	19.0	2.7	7.1	14.7	21.3	3.4	4.6	8.5
Beauvericin	35.0	6.5	66.3	309.8	50.8	3.0	10.4	20.7
Alternariol	40.7	5.0	15.4	69.8	8.2	5.0	5.0	5.0
Moniliformin	20.8	4.1	30.0	77.6	14.8	3.5	12.1	12.3
Sterigmatocystin	16.8	1.5	7.7	16.9	8.2	1.2	2.3	3.0
Mycophenolic acid	11.9	5.6	13.8	24.7	6.6	10.4	20.3	23.3
Penicillic acid	0.9	12.5	12.5	12.5	Not detected.			
Roquefortine C	0.4	14.4	14.4	14.4	Not detected.			
**Ergot alkaloids**								
Ergocryptine	8.4	7.3	16.1	17.9	27.9	7.5	45.5	96.0
Ergocryptinine	6.2	7.5	20.5	27.1	26.2	18.3	46.5	50.5
Ergotamine	21.2	7.6	21.3	27.2	26.2	10.2	28.9	33.0
Ergocristine	9.3	9.5	11.1	36.3	18.0	19.6	125.3	151.3
Ergocristinine	14.6	15.0	50.4	60.8	24.6	65.1	97.2	104.9
Ergosin	9.7	7.5	14.1	28.8	23.0	9.5	17.8	19.3
Ergocornine	5.3	7.1	7.5	18.1	21.3	7.9	30.0	34.6
Ergometrine	4.9	7.5	21.6	27.1	18.0	8.8	12.9	17.1
Ergometrinine	1.3	1.1	1.2	1.3	18.0	3.7	10.1	10.6
Agroclavine	4.0	1.3	2.6	3.0	3.3	0.5	0.5	0.5
Ergine	0.9	6.8	7.0	7.0	Not detected.			

^a^ Sum of fumonisins B_1_ and B_2_.

**Table 2:** Fractions of samples exceeding maximum levels or guidance values for mycotoxins in feed and feed raw materials that are in effect in the European Union.

	Aflatoxin B_1_	Fumonisins B_1_ + B_2_ ^a^	Deoxynivalenol	Zearalenone	Ochratoxin A
Fish feed ^b^	7.1%	0.4%	0.0%	0.0%	0.0%
Shrimp feed ^b^	1.6%	0.0%	0.0%	0.0%	0.0%
Maize ^c^	5.0%	0.0%	0.2%	0.1%	0.1%
Maize DDGS ^d^	0.7%	0.0%	0.7%	0.0%	0.0%
Wheat ^e^	0.0%	0.0%	1.3%	0.1%	0.0%
Soybean ^e^	0.9%	0.0%	0.0%	0.0%	0.0%
Rice ^e^	4.6%	0.0%	0.0%	0.0%	0.0%

^a^ Sum of fumonisins B_1_ and B_2_. ^b^ EU limits applicable for fish and shrimp feed: maximum level for aflatoxin B_1_—10 µg/kg; guidance value for fumonisins B_1_ + B_2_—10 mg/kg; guidance value for deoxynivalenol—5 mg/kg; guidance value for zearalenone—2 mg/kg; guidance value for ochratoxin A—250 µg/kg. ^c^ EU limits for maize: maximum level for aflatoxin B_1_—20 µg/kg; guidance value for fumonisins B_1_ + B_2_—60 mg/kg; guidance value for deoxynivalenol—8 mg/kg; guidance value for zearalenone—2 mg/kg; guidance value for ochratoxin A—250 µg/kg. ^d^ EU limits for maize DDGS: maximum level for aflatoxin B_1_—20 µg/kg; guidance value for fumonisins B_1_ + B_2_—60 mg/kg; guidance value for deoxynivalenol—12 mg/kg; guidance value for zearalenone—3 mg/kg; guidance value for ochratoxin A—250 µg/kg. ^e^ EU limits for wheat, soybean, and rice: maximum level for aflatoxin B_1_—20 µg/kg; guidance value for fumonisins B_1_ + B_2_—10 mg/kg; guidance value for deoxynivalenol—8 mg/kg; guidance value for zearalenone—2 mg/kg; guidance value for ochratoxin A—250 µg/kg.

**Table 7:** Mycotoxin occurrence in maize and maize DDGS.

	Maize (n = 3448)				Maize DDGS (n = 149)			
**Mycotoxin**	Prevalence (% Positive Samples)	Median of Positives (µg/kg)	90th Percentile of Positives (µg/kg)	Maximum (µg/kg)	Prevalence (% Positive Samples)	Median of Positives (µg/kg)	90th Percentile of Positives (µg/kg)	Maximum (µg/kg)
**Regulated mycotoxins (EU maximum level or guidance value)**								
Aflatoxin B_1_	14.4	7.2	112.0	2257.5	26.2	1.0	7.9	70.5
Fumonisins B_1_ + B_2_ ^a^	73.3	422.0	3193.9	55,403.0	97.3	663.8	1867.1	10,614.2
Deoxynivalenol	70.4	319.6	1622.7	42,449.0	92.6	2114.4	4472.4	15,027.4
Zearalenone	53.7	17.3	141.3	9454.8	96.0	169.9	491.5	2328.5
Ochratoxin A	3.2	2.1	43.6	571.0	20.8	2.1	7.6	39.0
**Other aflatoxins**								
Aflatoxin B_2_	5.9	3.2	17.2	237.7	4.7	1.5	8.1	14.9
Aflatoxin G_1_	2.0	4.6	21.0	351.1	2.0	1.5	1.5	1.5
Aflatoxin G_2_	0.4	1.5	7.9	56.2	Not detected			
**Fumonisins**								
Fumonisin B_1_	72.2	322.8	2427.1	39,916.8	95.3	498.2	1461.9	6609.0
Fumonisin B_2_	67.8	124.6	814.1	15,486.2	94.0	180.3	501.0	4005.2
Fumonisin B_3_	58.4	63.1	336.7	4978.5	89.9	74.8	203.0	1828.7
**Other trichothecenes**								
Deoxynivalenol-3-glucoside	54.9	71.1	297.4	10,606.7	83.2	252.9	683.7	1434.9
15-Acetyl-deoxynivalenol	34.2	101.3	356.0	5059.4	71.8	387.7	802.8	2146.1
3-Acetyl-deoxynivalenol	5.7	75.0	161.0	1099.8	25.5	75.0	187.1	633.0
Nivalenol	18.3	68.9	352.6	5357.8	18.8	30.0	175.2	230.0
T-2 toxin	6.6	15.0	97.8	904.3	38.9	15.0	61.8	176.9
HT-2 toxin	5.3	25.0	205.4	2299.9	43.0	25.0	160.7	398.5
T-2 triol	1.2	25.0	78.2	250.0	5.4	15.0	49.6	86.8
T-2 tetraol	0.6	57.7	350.0	1567.2	0.7	15.2	15.2	15.2
Monoacetoxyscirpenol	2.7	7.6	16.6	116.6	2.0	15.0	15.0	15.0
Diacetoxyscirpenol	4.0	5.0	10.4	41.4	4.7	5.0	5.0	5.0
Neosolaniol	2.9	3.2	11.7	57.9	6.7	2.5	7.4	13.9
Fusarenon-X	0.8	50.0	154.6	492.9	1.3	50.0	50.0	50.0
**Zearalenone metabolites**								
beta-Zearalenol	3.1	10.0	28.2	330.6	71.1	56.3	169.0	624.9
alpha-Zearalenol	2.1	5.0	14.7	89.4	45.6	11.7	27.4	83.2
**Other ochratoxins**								
Ochratoxin B	1.3	1.0	9.7	32.2	6.0	1.0	2.0	4.2
**Emerging mycotoxins (less investigated fungal metabolites)**								
Enniatin B	18.4	1.5	28.5	5043.7	51.0	2.8	65.9	271.5
Enniatin B1	15.1	1.5	16.4	827.0	29.5	5.2	43.9	144.6
Enniatin A	5.9	1.3	4.8	31.7	16.8	1.5	3.9	7.8
Enniatin A1	9.0	1.5	9.4	148.4	16.8	5.2	20.6	60.4
Beauvericin	54.2	7.7	108.9	1723.3	87.2	26.2	202.6	1082.8
Moniliformin	62.0	132.2	638.8	7127.9	87.2	124.0	459.8	2095.9
Alternariol	18.7	6.3	37.5	1588.5	55.7	15.0	30.2	94.5
Sterigmatocystin	7.1	0.5	4.0	148.4	44.3	0.5	3.7	235.2
Mycophenolic acid	5.0	25.0	214.4	7042.0	30.9	30.0	151.4	935.7
Patulin	0.1	75.0	307.1	365.1	3.4	125.0	125.0	125.0
Roquefortine C	0.8	6.2	412.8	1588.6	4.0	3.0	6.5	9.9
Penicillic acid	1.5	40.9	180.9	1404.7	Not detected			
Gliotoxin	0.2	75.0	191.1	307.1	Not detected			
**Ergot alkaloids**								
Ergocryptine	0.1	2.5	5.6	6.9	4.0	6.9	21.5	30.7
Ergocryptinine	0.5	4.6	9.9	14.0	1.3	7.0	10.3	11.1
Ergotamine	0.1	7.2	7.3	7.3	2.7	18.3	30.5	31.9
Ergometrine	0.7	1.5	16.3	40.5	4.0	4.3	9.8	11.5
Ergometrinine	0.1	1.5	11.6	15.9	0.7	9.5	9.5	9.5
Ergocornine	0.3	1.5	18.1	21.5	3.4	5.2	8.3	9.1
Ergocristine	0.1	7.5	17.8	20.6	3.4	12.5	37.3	53.2
Ergocristinine	0.4	3.0	19.4	22.0	3.4	6.3	13.2	15.0
Ergine	Not detected				2.7	2.0	2.9	3.0
Ergosin	0.2	4.5	11.7	15.8	2.7	14.0	22.0	22.6
Agroclavine	0.3	0.5	2.5	7.3	0.7	1.2	1.2	1.2
Dihydrolysergol	0.1	25.0	25.0	25.0	Not detected			
Elymoclavine	0.03	2.0	2.0	2.0	Not detected			

^a^ Sum of fumonisins B_1_ and B_2._

**Table 8:** Mycotoxin occurrence in wheat, soybean, and rice.

	Wheat (n = 1578)				Soybean (n = 428)				Rice (n = 65)			
	Prevalence (% Positive Samples)	Median of Positives (µg/kg)	90th Percentile of Positives (µg/kg)	Maximum (µg/kg)	Prevalence (% Positive Samples)	Median of Positives (µg/kg)	90th Percentile of Positives (µg/kg)	Maximum (µg/kg)	Prevalence (% Positive Samples)	Median of Positives (µg/kg)	90th Percentile of Positives (µg/kg)	Maximum (µg/kg)
**Regulated mycotoxins (EU maximum level or guidance value)**												
Aflatoxin B_1_	0.9	1.0	4.9	5.7	7.2	1.1	32.3	84.0	24.6	3.5	24.3	76.4
Fumonisins B_1_ + B_2_ ^a^	13.0	15.0	113.4	1402.1	22.9	30.0	100.4	1672.1	81.5	30.0	127.0	623.2
Deoxynivalenol	68.9	115.7	1289.4	22,984.2	11.9	30.1	557.1	679.3	30.8	30.0	32.4	383.8
Zearalenone	29.1	12.5	82.1	14,049.0	32.7	12.5	53.8	1349.3	61.5	37.6	153.4	954.6
Ochratoxin A	3.4	2.0	9.5	161.8	6.8	1.0	16.2	36.6	9.2	1.8	4.6	5.5
**Other aflatoxins**												
Aflatoxin B_2_					1.2	4.3	7.5	8.8	4.6	1.5	4.6	5.4
Aflatoxin G_1_	0.1	3.3	3.3	3.3	0.7	4.6	5.2	5.3	Not detected			
**Fumonisins**												
Fumonisin B_1_	7.1	15.0	138.1	1121.3	14.5	15.0	76.9	1284.0	49.2	39.2	122.5	475.8
Fumonisin B_2_	11.3	15.0	42.5	280.8	18.0	15.0	60.3	388.1	80.0	15.0	40.1	147.4
Fumonisin B_3_	4.8	15.0	33.2	158.4	6.1	15.0	40.2	256.8	44.6	15.0	15.0	69.2
**Other trichothecenes**												
Deoxynivalenol-3-glucoside	50.3	25.0	244.4	1654.9	3.3	7.5	57.0	162.4	3.1	50.2	75.9	82.3
15-Acetyl-deoxynivalenol	6.8	28.1	296.1	634.8	0.5	75.0	75.0	75.0	32.3	75.0	75.0	75.0
3-Acetyl-deoxynivalenol	6.5	15.4	124.4	218.4	2.1	75.0	214.5	409.2	Not detected			
Nivalenol	32.5	30.0	220.5	3443.2	4.2	92.0	147.0	168.5	9.2	96.1	128.0	138.9
T-2 toxin	7.3	3.2	15.1	103.9	1.9	15.0	79.0	148.6	1.5	2.0	2.0	2.0
HT-2 toxin	11.2	12.9	26.7	184.6	1.9	46.3	233.0	375.9	1.5	7.1	7.1	7.1
T-2 triol	0.1	26.7	36.0	38.3	Not detected				Not detected			
T-2 tetraol	1.4	14.0	150.0	150.0	0.5	150.0	150.0	150.0	Not detected			
Monoacetoxyscirpenol	4.1	6.0	15.0	79.9	1.2	15.0	34.9	48.1	15.4	15.0	36.7	43.5
Diacetoxyscirpenol	0.5	2.5	6.3	9.3	0.7	2.2	4.4	5.0	15.4	5.0	11.8	20.2
Neosolaniol	1.3	3.1	10.4	15.9	0.7	1.9	5.7	6.6	Not detected			
Fusarenon-X	0.3	50.0	50.0	50.0	Not detected				Not detected			
**Zearalenone metabolites**												
beta-Zearalenol	2.2	5.6	32.9	50.6	2.6	10.0	98.6	132.7	6.2	10.0	18.3	21.8
alpha-Zearalenol	1.7	5.0	18.9	38.5	4.0	5.0	83.8	228.6	4.6	5.0	5.0	5.0
**Other ochratoxins**												
Ochratoxin B	0.3	1.0	3.7	5.5	1.9	5.8	12.5	13.8	3.1	1.0	1.0	1.0
**Emerging mycotoxins (less investigated fungal metabolites)**												
Enniatin B	57.9	16.7	149.0	3996.3	45.3	1.5	31.2	2612.8	3.1	41.9	74.1	82.2
Enniatin B1	71.8	16.6	109.6	1304.5	43.9	1.5	13.6	1766.2	3.1	10.8	18.2	20.1
Enniatin A	33.0	2.1	8.3	31.8	13.8	1.5	5.7	102.6	1.5	1.5	1.5	1.5
Enniatin A1	58.5	6.6	35.3	218.9	26.6	1.5	11.7	540.9	3.1	2.4	3.1	3.3
Beauvericin	38.2	3.0	12.4	285.4	62.4	3.2	22.7	170.3	36.9	3.4	31.8	102.5
Moniliformin	40.5	15.0	103.5	1311.8	16.8	8.7	33.0	98.9	58.5	40.1	126.0	480.5
Alternariol	12.6	15.0	143.2	4841.6	13.8	9.4	48.1	264.0	4.6	7.7	13.5	15.0
Sterigmatocystin	4.1	0.5	4.9	72.3	9.3	0.5	2.3	10.5	67.7	0.5	3.4	77.9
Mycophenolic acid	1.8	15.0	81.1	157.0	1.4	27.5	98.8	127.9	Not detected			
Patulin	0.6	125.0	151.2	386.7	0.2	125.0	125.0	125.0	Not detected			
Roquefortine C	0.5	10.9	23.2	27.5	Not detected				Not detected			
Penicillic acid	0.1	12.5	12.5	12.5	Not detected				Not detected			
**Ergot alkaloids**												
Ergocryptine	10.0	12.2	131.6	580.4	1.4	2.8	33.6	59.8	Not detected			
Ergocryptinine	6.7	7.5	32.7	169.6	1.6	2.9	6.2	7.5	Not detected			
Ergosin	10.4	9.6	143.7	1216.5	0.2	22.4	22.4	22.4	Not detected			
Ergocristine	10.0	13.8	270.6	2362.6	0.9	14.7	686.8	970.5	Not detected			
Ergocristinine	6.7	9.4	128.0	523.9	0.9	16.6	55.6	68.1	Not detected			
Ergotamine	8.2	21.7	245.3	1351.9	1.2	4.1	187.2	307.4	Not detected			
Ergocornine	7.4	15.7	152.3	498.0	0.2	52.4	52.4	52.4	Not detected			
Ergometrine	6.5	7.7	47.0	326.7	0.2	94.0	94.0	94.0	Not detected			
Ergometrinine	4.8	3.9	29.9	706.2	0.2	13.1	13.1	13.1	Not detected			
Agroclavine	1.2	1.3	4.1	4.6	0.7	1.0	9.5	11.6	3.1	0.5	0.5	0.5
Ergine	0.4	1.5	4.6	6.6	0.9	1.5	4.9	6.3	Not detected			
Elymoclavine	0.1	1.6	2.0	2.1	0.2	1.5	1.5	1.5	Not detected			

^a^ Sum of fumonisins B_1_ and B_2._

**Table 9:** Limits of detection, limits of quantification, and apparent recovery as reported by Sulyok et al. [142,143].

Mycotoxin	LOQ ^a^ (µg/kg)	LOD ^b^ (µg/kg)	Apparent Recovery (%)
15-acetyl-deoxynivalenol	30.6	9.2	89
3-acetyl-deoxynivalenol	16.0	4.8	64
Aflatoxin B_1_	0.7	0.2	95
Aflatoxin B_2_	0.2	0.06	58
Aflatoxin G_1_	0.5	0.2	85
Aflatoxin G_2_	1.7	0.5	68
Agroclavine	0.3	0.1	93
α-Zearalenol	3.8	1.1	71
Alternariol	0.3	0.1	46
Beauvericin	0.08	0.03	110
β-Zearalenol	9.8	2.9	71
Deoxynivalenol	3.9	1.3	111
Deoxynivalenol-3-glucoside	4.2	1.4	102
Diacetoxyscirpenol	0.5	0.1	76
Dihydrolysergol	0.4	0.1	89
Elymoclavine	0.9	0.3	94
Enniatin A	0.03	0.01	89
Enniatin A_1_	0.2	0.06	91
Enniatin B	0.04	0.01	91
Enniatin B_1_	0.1	0.03	80
Ergine	0.2	0.07	82
Ergocornine	1.2	0.4	52
Ergocristine	1.2	0.4	46
Ergocristinine	0.9	0.3	62
Ergocryptine	1.4	0.4	58
Ergocryptinine	1.1	0.3	69
Ergometrine	3.6	1.1	119
Ergometrinine	0.1	0.03	89
Ergosine	1.4	0.4	85
Ergotamine	3.1	0.9	78
Fumonisin B_1_	8.0	2.4	55
Fumonisin B_2_	7.1	2.1	62
Fumonisin B_3_	19.2	5.8	64
Fusarenon X	12.9	3.9	84
Gliotoxin	4.0	1.2	65
HT-2 toxin	5.0	1.5	82
Moniliformin	5.1	1.5	83
Monoacetoxyscirpenol	5.3	1.6	83
Mycophenolic acid	3.7	1.1	96
Neosolaniol	2.9	0.9	56
Nivalenol	2.6	0.8	73
Ochratoxin A	1.5	0.5	88
Ochratoxin B	0.4	0.1	86
Patulin	1933.0	579.9	8
Penicillic acid	1.5	0.4	53
Roquefortine C	1.6	0.5	64
Sterigmatocystin	0.3	0.08	104
T-2 tetraol	9	3	70
T-2 toxin	2.4	0.7	97
T-2 triol	11.9	3.6	83
Zearalenone	0.6	0.2	85

^a^ Limit of quantification, ^b^ Limit of detection.

## Data Availability

The original contributions presented in this study are included in this article. Further inquiries can be directed to the corresponding author.
